# PsychAdapter: adapting LLMs to reflect traits, personality, and mental health

**DOI:** 10.1038/s44387-026-00071-9

**Published:** 2026-03-02

**Authors:** Huy Vu, Huy Anh Nguyen, Adithya V. Ganesan, Swanie Juhng, Oscar N. E. Kjell, Joao Sedoc, Margaret L. Kern, Ryan L. Boyd, Lyle Ungar, H. Andrew Schwartz, Johannes C. Eichstaedt

**Affiliations:** 1 Computer Science Department, Stony Brook University, Stony Brook, NY USA; 2 Stern School of Business, New York University, New York, NY USA; 3 Centre for Wellbeing Science, University of Melbourne, Melbourne, VIC Australia; 4 Department of Psychology, University of Texas at Dallas, Richardson, TX USA; 5 Computer and Information Science, University of Pennsylvania, Philadelphia, PA USA; 6 College of Connected Computing, Vanderbilt University, Nashville, TN USA; 7 Psychology Department & Institute for Human-Centered AI, Stanford University, Stanford, CA USA; 8 Decision Sciences, INSEAD, Fontainebleau, Paris France

**Keywords:** Mathematics and computing, Psychology, Psychology

## Abstract

AI language generators are now ubiquitous but typically produce generic text that fails to reflect individual differences. Here, we introduce PsychAdapter, a lightweight LLM architectural modification that uses empirically derived links between language and personality, demographic, and mental health traits to generate trait-reflective text, regardless of prompt. PsychAdapter was applied to GPT-2, Gemma-2B, and LLaMA-3, and expert raters confirmed that the generated text matched the specified traits: it produced Big Five personality traits with 87.3% and depression and life satisfaction with 96.7% accuracy. PsychAdapter is a novel method for embedding psychological behavioral patterns into language models by conditioning every transformer layer, without relying on prompting. Beyond personality-conditioned generation, this approach has potential uses for simulated patients reflecting psychopathology and translation tailored to reading or educational level. It also enables generation of characteristic sentences for studying the language of traits, expanding the language processing toolkit for psychology.

## Introduction

The *transformer language model*^1,2^ is a paradigm-shifting technique in Artificial Intelligence (AI) that has been integrated into everyday applications, including web search, content recommendations, and question-answering. These *transformers*, which are behind most large language models, including ChatGPT^2^, Gemma^3^, and LLaMA^4^, can generate text that is strikingly similar to natural human language^5^. However, the generated text represents average patterns aggregated across many documents with corresponding authors, reflecting a limited range of expressed psychological attributes^6–8^. The models do not explicitly represent differences in human traits – the fundamental characteristics that distinguish people^9^, for which decades of research have demonstrated that language use patterns vary widely^10,11^.

**Fig. 1:**
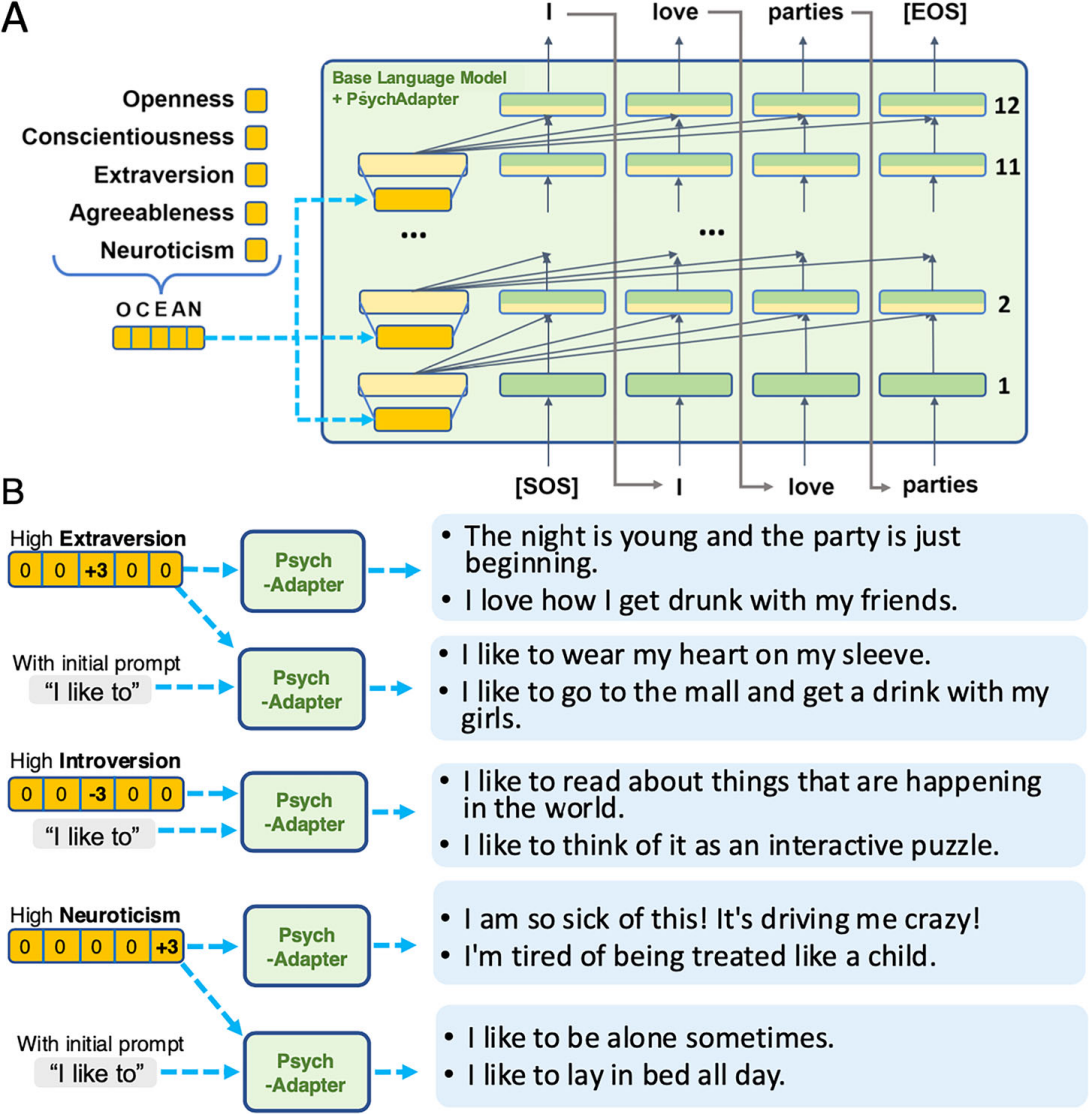
PsychAdapter architecture and inferencing procedure. **A** Overview of PsychAdapter augmenting the widely-used auto-regressive transformers architecture^2^ to incorporate personality scores (empirically associated with text) as additional inputs together with prior words to learn to reconstruct text. Personality scores are continuous real numbers (e.g., -1.3, 0.5) that roughly reflect standard deviations above or below the population mean for those traits. **B** After training, the base language model augmented with PsychAdapter can take different inputs reflecting any specific personality pattern and generate text accordingly. The output text can be prompted with initial words, such as *"I like to''* to successfully complete the sentence according to different individual characteristics. Two special tokens, “[SOS]” (start-of-sentence) and “[EOS]” (end-of-sentence), signal the start and end points of text generation.

Here we present *PsychAdapter* (source code can be found at: https://github.com/humanlab/psychadapter.), a lightweight augmentation to any auto-regressive transformer language model, the standard machine learning architecture behind most modern Large Language Models (LLMs), such as GPT, Gemma, and LLaMA, to produce language reflective of individual psychological characteristics. PsychAdapter was initially trained to cover the Big Five personality traits (openness, conscientiousness, extraversion, agreeableness, and neuroticism) as well as mental health variables (depression and life satisfaction), while simultaneously being conditioned on demographics (e.g., age or gender). It generates text that reflects authors scoring high or low in any of these factors, and in any combination. For example, it can produce text characteristic of extraverts by setting extraversion = +3 (roughly, standard deviations above the population mean), or that of a young person who is depressed (depression = +3, age = -3). Like all generative language models, PsychAdapter can continue sentences after a prompt, for instance, illustrating how a person with high neuroticism would complete “I hate it when” or “I like to” (see Fig. 1). Our study shows that such prompts can foreground token generation that is particularly relevant to personality or well-being. We evaluated PsychAdapter using both human raters with psychology training and large language models (e.g., claude by anthropic^12^) to assess how well the intended trait characteristics can be inferred from their outputs.

Equipping AI transformers with demographics and psychological traits offers a range of potential applications. For example, PsychAdapter could enable the development of chatbots with more diverse and human-like personalities. Customer service staff could be trained with these systems mimicking customers with different personalities and emotional states. New crisis line and mental health responders could be trained, without risk to patients, using simulated conversation partners expressing different levels of depression and personality characteristics, aiding in their ability to pick up indications of distress without high-risk patient interactions. Further, transformer-based text generation models are built into many modern applications, and thus our proposed modifications can propagate to improving their standard applications such as machine translation or personalized assistants. For example, answers could be generated based on matching different education, dialect, or age levels to be more accessible to different audiences. By adjusting trait scores, PsychAdapter presents new degrees of freedom to enable more diverse human-like language generation.

For researchers, PsychAdapter can be seen as a new type of *differential language analysis* (DLA) – a technique that empirically elicits the language that differentiates psychological constructs^13–16^. Prior approaches to DLA suffer from a lack of context^11^. PsychAdapter addresses this by generating characteristic coherent sentences of traits rather than discrete words or phrases that are more ambiguous (e.g., ‘play’ in “I am constantly being played" in neuroticism versus “A date night with my spouse is play time” in high extraversion). More context enables more robust interpretations and higher-quality synthetic data for further use.

Our work builds upon a progression of research using statistical and machine learning to investigate the connections between personality traits, mental health, and human language^13,14,17–20^. However, our approach advances this line of work by generating fully formed text that captures rich contextual information, rather than foregrounding abstract, decontextualized displays of words, phrases or topics associated with psychological dimensions. Our work also builds on previous studies in text generation that express speakers’ and writers’ psychological traits^21–30^. However, these works emphasize developing personalized dialogue models that are conditioned on speakers’ personas represented by discrete attributes values like age, gender, region, or self-described statements, instead of continuous personality scores as in our work. Continuous psychological scores as input potentially provide better control over levels of language expression and can simulate near-infinite psychological profiles.

To implement this idea, PsychAdapter directly modifies the underlying transformer architecture, drawing on empirically derived personality-language associations^13,18^ rather than relying on prompt-based control. Figure 1A summarizes the architecture of PsychAdapter, which extends the generative transformer language model to incorporate personality factors as input. PsychAdapter builds on work in AI for conditional language modeling^2–4^. However, instead of conditioning only on text, it enables input of continuous dimensional psychological traits, such as personality or mental health variables, and outputs natural language that reflects these characteristics. The input vector (represented as dark yellow in Fig. 1) can be a single psychological score or any combination of scores. Detailed in *materials and methods*, our modified transformer architecture can also condition on an input list of psychological scores through a learned dimension expansion per transformer layer, enabling the psychological scores to influence the generative process at every transformer layer. Just like standard generative language models, PsychAdapter is trained with the objective of best predicting the next word, but instead of just learning weights for the transformer itself, it also learns how to weigh the psychological scores’ contribution to each layer.

We trained and validated multiple transformer language models with PsychAdapter using a dataset of open-source public social media and blog posts along with an empirical model that estimates the Big Five personality scores for a given text document^18^. After training, the PsychAdapter was queried to produce text conditioned on vectors of Big Five (Openness, Conscientiousness, Extraversion, Agreeableness, and Neuroticism) personality scores. To *instruct* the model to generate text distinguishing a particular psychological attribute, we set its psychological score to a high value ( + *k* × *σ*_*i*_) and the other dimensions to their mean value (*μ*_*j*_, with *i* ≠ *j*). For example, if we want generated text to reflect *extraversion*, which is the third dimension of the input Big Five vector, we would feed the following vector into the model: (*μ*_*O*_, *μ*_*C*_, *μ*_*E*_ + *k*. *σ*_*E*_, *μ*_*A*_, *μ*_*N*_), with *k* being any value from the range [ − 3, 3] – akin to a 7-point Likert scale used in psychological surveys. We designed PsychAdapter to work with normalized trait scores (*μ*_*i*_ = 0, *σ*_*i*_ = 1), hence, we would use (0, 0, + *k*, 0, 0) as input for the previous example. This enables simultaneous control over all dimensions; the model can be set to produce text corresponding to a combination of different scores by adjusting the input Big Five vector, such as placing a high value on one dimension and a low value on another. For example, the input (*O*, *C*, *E*, *A*, *N*) = ( + 3, 0, − 3, 0, 0) will generate text having both high openness and low extraversion while being average in the other three dimensions.

Once trained, thanks to the small size of PsychAdapter (added parameters are less than 0.1% of original base language models: e.g. for Gemma 2B model, 55,296 parameters added compared to 2 billion parameters of base model), an adapter can be easily distributed to be used with the base model. These lightweight “adapters” (each adapter corresponds to a different set of psychological or demographic variables) equip the base language models with the capability to generate text with fine-grained control of underlying psychological profiles. This benefit of PsychAdapters is similar to the benefits of Parameter-Efficient Fine-Tuning (PEFT)^31^ methods, which enable language models with fine-tuning capabilities by adding few parameters to the base model.

## Results

### Generating text for personality dimensions

We first explored conditioning PsychAdapter on a single personality trait by specifying a high or low score ( + 3/ − 3 points) for the trait of interest, while assigning scores of 0 (i.e., the mean) in all other dimensions. Note that while we set the score values to integers here to illustrate PsychAdapter’s capability, in practice, these score values can be any floating-point numbers on the continuous personality dimension. Figure 1B shows text generated for high and low extraversion and neuroticism. As might be expected based on the traits, PsychAdapter produced language related to friends and social activities for high extraversion, while it generated references to solitary activities for low extraversion and expressions of neurosis for high neuroticism. Additionally, as an auto-regressive language model, PsychAdapter can be prompted with initial words to complete the rest of the sentence. We can leverage this capability to generate text corresponding to specific topics. In Fig. 1B, we illustrate this approach by prompting PsychAdapter with *“I like to”* to reflect the interests or hobbies of people with different personalities. Table 1 shows selected examples of generated text from PsychAdapter for the dimensions of agreeableness and extraversion. Table 2 further explores other prompts that illustrate different language expression with specific personalities. Additional randomly selected generated examples for other personality dimensions, with and without the prompt *“I like to,”* can also be found in the S1 section of Supplementary Information.

**Table 1:** Selected text generated by PsychAdapter (based on Gemma-2B) for different personalities and mental health states

Dim.	High (+3)	Low (-3)
**Extraversion**	• the night is young and the party is just beginning • i’m ready for some good sex tonight. • i love how i get drunk with my friends • i’m so excited! tonight’s my 20th birthday! woohoo! • i’m excited for my trip to new york.	• the only reason i’m still playing the sims is to get the new expansion pack • the weather in my city is so nice today. • i finally got the computer fixed.i think it was a loose connection on the power supply. • i found a book i read a long time ago, but i never finished, and i thought it might be time to finish it • the computer is back up and running.
**Extraversion("i like to”)**	i like to.... party and drink with my friends... wear my heart on my sleeve... go to the mall and get a drink with my girls.. wear a lot of make up and have a lot of hair.. dress up and make my boyfriend smile.	i like to read a lot.i read books in my free time, and i’ve been doing it since i was a kid... read about things that are happening in the world use my computer to play games.. watch movies and read books be normal, i don’t like to be different.
**Agreeableness**	• i’m glad i have my best friend by my side. • i’ll never forget you, i’ll never forget you, i’ll never forget you, i’ll never forget you. • have a new friend. His name is j. and he’s awesome. • i am so excited about this weekend. it’s going to be so much fun. i love you all, and can’t wait to see you all on saturday. • my heart just felt the love jesus!	• i know you’re f***ing with me. i’m a f***ing idiot. • i hate when people talk to you like you’re a baby. • i’m so f***ing mad at my mom right now.i just got home from my motherf***in’ job and i get a call from her ass.i don’t f***ing want to talk to her right now. • this is the stupidest thing i’ve ever seen. • i swear people be talking crazy.
**Lifesatisfaction**	• i’m a simple man with simple desires • well, i had a pretty good weekend.i did nothing but lay on my couch and watch tv and play xbox.i’m pretty sure i’ll do that again this weekend. • today’s a pretty good day for me. • my boyfriend is so cute! he makes me happy! • i’m so excited for my wedding.	• i hate when people are fake and shit. • i’m tired of this bullshit. i’m tired of being treated like a child. • i hate when people talk to me with their mouth full • i’m sick of this bullshit. i need to move on. • i’m so sick of this damn school.
**Depression**	• my dad is really getting on my nerves • i feel so lonely and sad today. • i feel so alone in this world • i hate when my mom gets angry and screams • i’m so f**king tired. my head hurts so f***ing bad.	• is in a good mood! • i have to say, it was a great day! • i’m back from vacation. it’s been a good vacation. we went to the beach and went to the amusement park. it was pretty fun. i’m glad I went. i’m glad I went. • the best thing about summer is summer school • i have the best girlfriend in the world.

The full set of random selected examples can be found in the S1 section of Supplementary Information.

**Table 2:** Selected text generated by PsychAdapter (based on Gemma-2B) for mental health variables with specified age

Dim.	Low Age (-3)	High Age (+3)
**High (+3)Depression**	• my brother is so annoying • why are people so stupid? • i’m so tired of my dad’s music. • my dad is such a bitch. • so i’m really really tired.and i’m so sick of being tired.	• i’m so tired of hearing my neighbors kids screaming and yelling. • i’m still sick, and i’ve been sick for 4 days now.i’m tired and i’m not getting much sleep. • my daughter has been in the hospital for over a year now. she’s on a ventilator and a feeding tube. • i hate when people treat me like i’m a child. • i hate to see the kids sad.
**Low (-3)Depression**	• my lil bro is the funniest kid ever • i’m ready for my lil girl to come home. • the fact that i love him makes me crazy • my lil bro is so funny lol • today’s the first day of school for me.it’s pretty exciting i’ll be starting a new class, which means i get to meet 10 new people.i’m excited, but also nervous.	• the weather was absolutely perfect for a walk yesterday. the sun was shining, the temperature was a perfect 60 degrees, and the air was fresh and clean. • my son is a good boy! • i’m thinking about getting another dog. • i am so ready to get home and get into my comfy ass clothes
**High (+3)Life satisfaction**	• so i went shopping today with my parents.it was fun!i got a really cute dress for my birthday! • well my first day of school was pretty fun. • i’m excited.i’ll be leaving for college in 13 days. • i’ll be leaving soon.i’m going to my cousin’s house in new york for a week. • i’ll be back the 8th of july.	• i love my husband and my daughter. • my son is a good kid. he is a very happy 2 year old. he is very happy to be here, very happy to be with me. • i’m so happy for the new couple! • my son just bought me a new pair of crocs • the lord’s blessings are so great.
**Low (-3)Life satisfaction**	• my hair is so stupid. i hate it. • i hate my sister so much. • people be so fake on twitter • i’m so bored my sister’s not on the computer so i’m stuck here. • this is my first blog and i really hope that i don’t screw it up.i’m just bored right now, but i’m going to try to write something interesting.	• i hate when people get on my nerves • i’m tired of hearing all the bullshit. • i’m getting old and fat and i’m losing my mind • my back is killing me. i hate this job. • i’m sick as a dog.i can’t keep the kids out of the hospital.i’m not getting paid.i’m not feeling good.my head hurts.my throat hurts.i’m cold.

A full set of randomly selected examples can be found in the S1 section of Supplementary Information.

### Evaluations with human raters and Claude

**Fig. 2:**
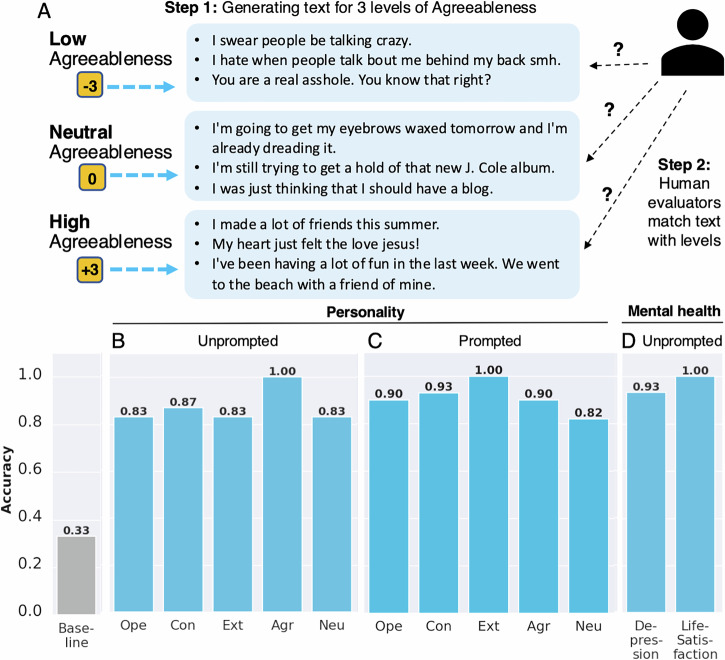
Overview of human expert evaluation set-up and results. **A** The set-up for human expert evaluation. For each variable, PsychAdapter generated a group of text from 3 input levels: Low, Neutral, and High. The blinded expert evaluator attempted to match the output text to the input level. **B–D** Results for human evaluation tasks, measured by accuracy as the percentage of correct matches, compared against the random-chance baseline (33.3%).

We evaluated the extent to which PsychAdapter’s output aligned with expert judgments using the setup illustrated in Fig. 2A. Specifically, for each personality factor *i*—such as agreeableness—we had the trained model generate a group of 5 samples for each of three input values of that dimension: Low, Neutral, and High, corresponding to *k* = − 3, *k* = 0, and *k* = 3, respectively. The blinded evaluators were then asked to read the generated text from the Low, Neutral, and High levels, presented in random order (5 samples per group, in line with the finding that multiple message samples are necessary to express personality^18^). Their task was to judge whether each group of messages corresponded to either the Low, Neutral, or High level. This process was repeated 10 trials for each dimension using 10 different random generating seeds, with 2 raters per task (both Ph.D.-level personality psychologists). Accuracy was calculated by determining how many of the evaluators’ classifications were correct across the 10 trials, using the average of the raters. Results were reported using a metric based on correctly distinguishing the three classes. If the evaluator average was correct for all three values, we assigned one “point” if correct for only one value (and incorrect for the other two), we assigned 1/3 of a point. These results were compared to a random chance baseline of 33.33%. Accuracies are reported in Fig. 2B. Across all dimensions, PsychAdapter significantly outperformed the random baseline of 33.3%, with an average accuracy of 87.3%. It performed better when prompted with “I like to” (Fig. 2C), which encouraged PsychAdapter to focus on activities typically associated with the trait, yielding an average accuracy of 91.0%. These results demonstrate PsychAdapter’s capability to generate text that reflects the input personality scores. In this evaluation, the weighted Cohen’s Kappa measuring agreement between two human experts is 0.76 (0.67 for without prompt and 0.85 for prompt “I like to”).

Besides using human experts to annotate the generated text by PsychAdapter, we also used the frontier LLM model Claude 3.5 Sonnet from Anthropic as a rater. This is a helpful approach for annotating extensive experiments that would require excessive human effort. Our findings indicate that annotation by Claude correctly identifies the intended traits with 93.5% accuracy for Big Five personality (compared to 89.2% by human annotators—averaged across no prompt and prompt “I like to”) and 100% Mental Health (compared to 96.7% by human annotators—averaged across depression and life satisfaction variable). We also find that Claude agrees with the human annotators at the same level (weighted Cohen’s Kappa of 0.81 for Big Five personality, and 0.95 for mental health, averaged across two human experts) as the human inter-rater agreement (weighted Cohen’s Kappa of 0.76 for Big Five personality, and 1.00 for mental health).

Based on these validations, we used Claude 3.5 Sonnet in subsequent subsections to annotate PsychAdapter’s text in extensive experiments that would have required a large amount of expert effort. Extensive details comparing LLM annotator and human annotators’ evaluations are reported in S2 section of Supplementary Information. In the following sections, unless specified, we employed Claude 3.5 Sonnet LLM for evaluating generated text from PsychAdapter.

### Generating text for mental health variables

To demonstrate the generalizability of our approach across psychological variables, we tested the proposed pipeline on mental health variables, specifically focusing on depression and life satisfaction. Using the same pipeline for the Big Five personality traits described in the Method section, we trained PsychAdapter to generate text for depression and life satisfaction variables. Table 1 presents selected examples generated by PsychAdapter for these two variables. Additional randomly selected examples can be found in the S1 section of Supplementary Information. Similar to Big Five personalities, we also evaluated this model through expert judgment, where two Ph.D.-level mental health experts served as raters, matching the generated text of undisclosed levels to three level classes of Low, Neutral and High. As shown in Fig. 2D, the human judges were able to distinguish the mental health text levels with an average accuracy of 96.67%.

### Generating text reflecting demographic profiles

Being able to adjust for demographic variables, and identify effects explained by more than such variables is important for many psychological studies. Control over demographic variables provides important speaker context when generating text as psychological processes may manifest differently in language depending on demographic traits, such as age and gender^10,32^. To explore this, we incorporated demographic information as an additional input for PsychAdapter, which was trained on mental health data described in the previous section. Specifically, we estimated age by applying an open-source model from prior research^33^, which assigns an estimated age score to each message. (This model^33^ has been shown to predict age within an average margin of error of 4 years of self-reported age.) The process is analogous to how we obtained the estimated Big Five personality scores, except in this case, the model predicts age instead of personality traits. We then appended the estimated age score to the psychological state vector, which here represents either a depression or life satisfaction score. The resulting input vector has two components: one for the mental health score (depression/life satisfaction) and one for age:

1 $$({\mu }_{1}+{k}_{1}\cdot {\sigma }_{1},\,{\mu }_{2}+{k}_{2}\cdot {\sigma }_{2})$$

where *μ*_1_ and *σ*_1_ are the mean and standard deviation of the depression or life satisfaction score, and *μ*_2_ and *σ*_2_ represent the mean and standard deviation of the estimated age score. The values *k*_1_ and *k*_2_ are scaling factors used to control the levels of the mental health and demographic variables. In the training step, this vector is fed into the model to reconstruct the original text, similarly to the illustration in Fig. 1A.

**Fig. 3:**
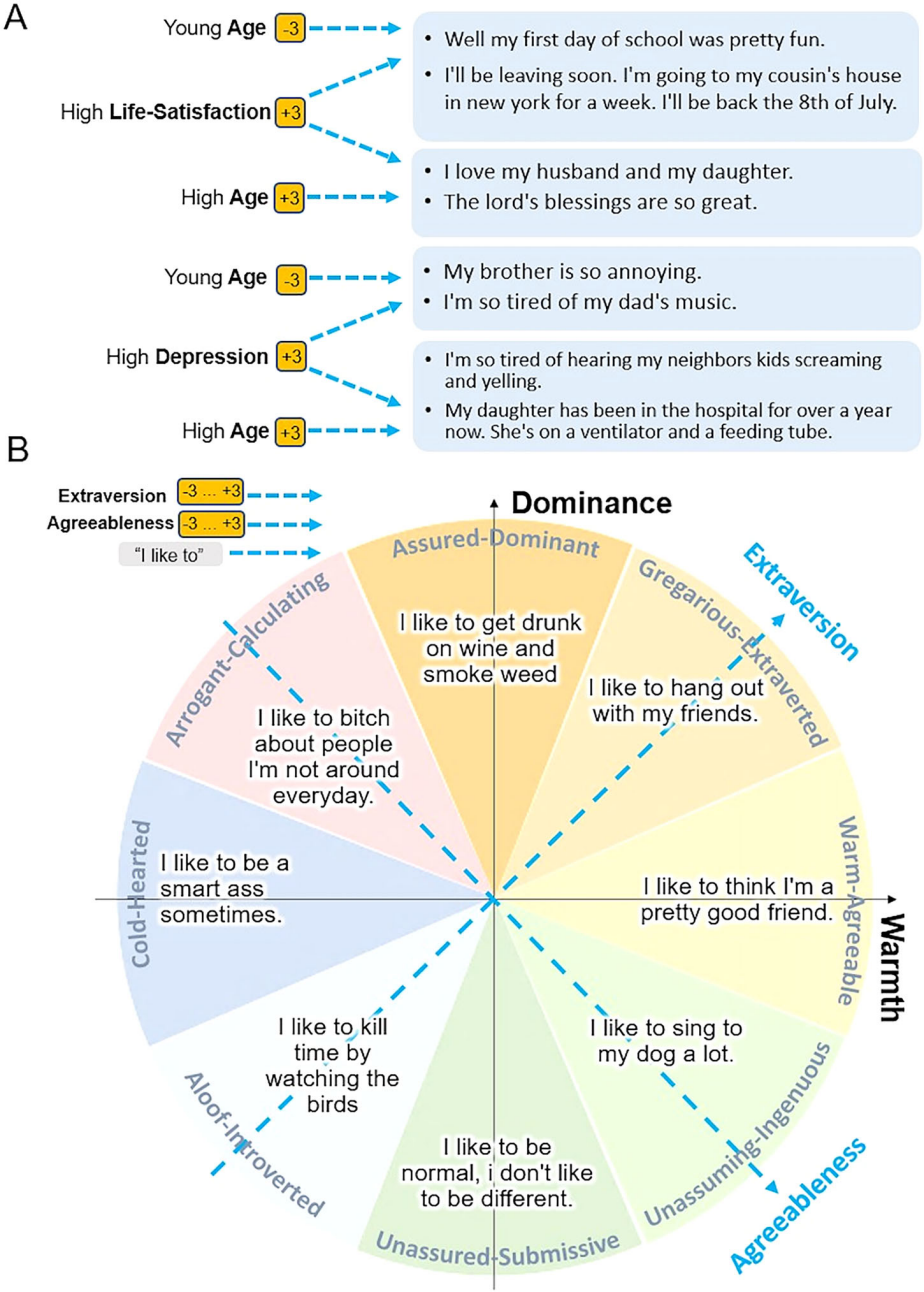
Controlling multiple dimensions in generation. **A** Generated text controlling both mental health variables (e.g., depression, life satisfaction) and demographics variables (e.g., age). **B** Text generated around the interpersonal circumplex. Extraversion and agreeableness can be rotated to yield values for dominance and warmth^72^ and, thus, positions in the circumplex. Text for the desired circumplex positions was generated by using the corresponding values of extraversion and agreeableness as inputs for PsychAdapter trained for Big5 personality based on Gemma-2B. Results show that generated language corresponds well to the intended theoretical structure of the circumplex.

After training, we can control both the mental health score and age when generating text. Figure 3A and Table 3 display selected examples of text generated for depression and life satisfaction while adjusting age. For both depression and life satisfaction, the generated text appears to correspond with the specified age. For instance, in the life satisfaction model, text conditioned on younger individuals referenced parents and school, while text for older individuals mentioned gratitude, spouses, and children. More randomly selected examples are provided in the S1 section of Supplementary Information. A human evaluation was conducted to assess the alignment of the generated text with underlying mental health and demographic variables. Specifically, PsychAdapter was conditioned on combinations of high and low values for both mental health scores and age - four combinations in total, and human experts were tasked with identifying the correct category for each set of generated texts. Results show that the two experts achieved 100% accuracy in this task, reflecting the capability of PsychAdapter to express both psychological and demographic variables simultaneously.

**Table 3:** Generalization across prompts: Selected text generated for Big Five personalities by Psych Adapter (based on Llama3-8B) base model, with prompts foregrounding different life and professional domains

### Generating text reflecting multiple personality dimensions

As described previously, PsychAdapter can generate text conditioned on multiple traits simultaneously by adjusting the input vector accordingly. We leveraged this capability to generate text based on the Interpersonal Circumplex^34,35^, a model used for organizing and assessing interpersonal behaviors, traits, and motives. The circumplex is defined by two axes, warmth and dominance, which can be understood as a rotation of the extraversion and agreeableness axes, with an angle *α* of 22.5 degrees^36^. The following equations express the mapping between the axes:

2 $$\begin{array}{rcl}{{\rm{score}}}^{Ext} & = & \cos (\alpha )\cdot {{\rm{score}}}^{Warmth}-\sin (\alpha )\cdot {{\rm{score}}}^{Dominance}\\ {{\rm{score}}}^{Agr} & = & \sin (\alpha )\cdot {{\rm{score}}}^{Warmth}+\cos (\alpha )\cdot {{\rm{score}}}^{Dominance}\end{array}$$

Using this relationship to map extraversion and agreeableness onto the warmth and dominance axes, we generated text using PsychAdapter with the corresponding Big Five input vector (0, 0, *score*^*Ext*^, *score*^*Agr*^, 0), positioning it within the interpersonal circumplex. The circumplex has previously been divided into segments with descriptors such as “Assured-Dominant” (high dominance, neutral warmth) or “Cold-Hearted” (neutral dominance, low warmth)^37^. As shown in Fig. 3B, we generated text using the prompt *“I like to”* in these different sections of the interpersonal circumplex, with the results conforming to theoretical expectations.

### Generating text at fine-grained levels of personality

One advantage of our method over prompt engineering, which relies on discrete token signals, lies in our ability to use continuous variable dimensions. This enables more precise and flexible control over the desired psychological profile (for example, it can precisely reflect a subject’s Big5 survey scores). To validate this, we conducted experiments in which PsychAdapter produced text at finer levels of granularity of psychological variable, specifically at: [ − 3 × *σ*_*i*_, − 1.5 × *σ*_*i*_, 0 × *σ*_*i*_, + 1.5 × *σ*_*i*_, + 3 × *σ*_*i*_]. The generated text was then evaluated to determine whether it matched the intended level of the psychological variable. For each personality dimension, we used PsychAdapter to generated text for five positions semantically corresponding to five levels: Very Low, Low, Neutral, High, and Very High. At each position, PsychAdapter generated ten samples. We repeat this ten trials with using different generating seed each time. Hence, for each personality dimension, PsychAdapter generated 5 positions × 10 samples × 10 trials.

**Fig. 4:**
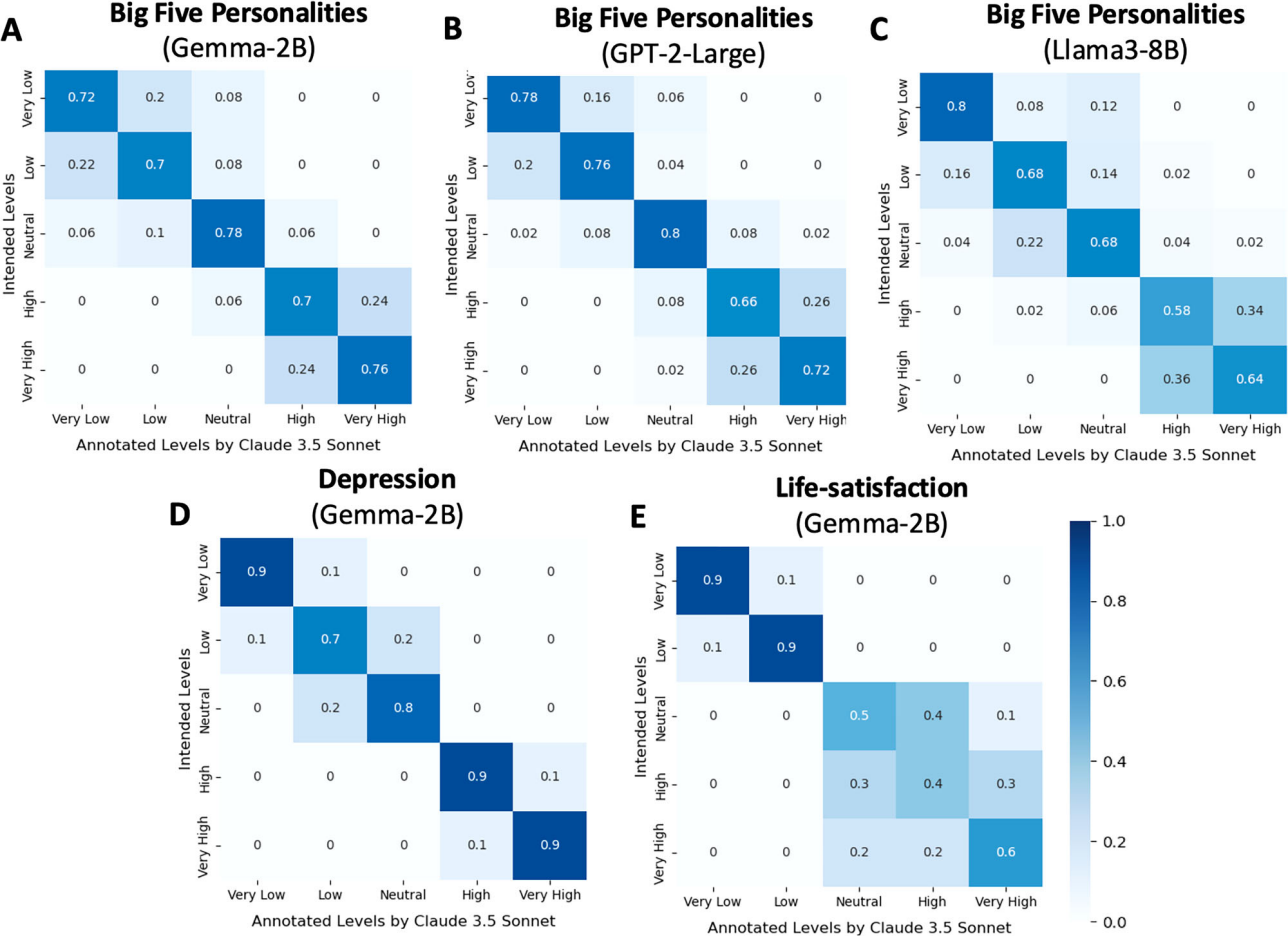
Accuracy of correspondence between five intended levels of PsychAdapter output annotations provided by Claude 3.5 Sonnet. **A** Big Five personalities with Gemma-2B; **B** GPT-2 Large, and **C** LLaMA3-8B; **D** Depression and **E** Life-satisfaction with Gemma-2B. Results are based on 10 generated samples per level and averaged across 10 different generation seeds. Results show that PsychAdapter is able to produce language reflective of nuanced differences in traits.

Following our previous demonstration that Claude 3.5 Sonnet performs similarly to human judges, we employed Claude as an automatic evaluator over this scaled-up evaluation set. Similar to the human judges in prior evaluations, the LLM was provided with the generated texts blinded to the five levels and tasked with annotating each test set for the intended level. The template of the prompt used for the LLM annotator is included in the S3 section of Supplementary Information. The results, presented in Fig. 4, show that PsychAdapter’s text generation, as annotated by Claude, aligns relatively well with the intended psychological levels at fine-grained resolutions. This highlights PsychAdapter’s ability to use a vector of continuous (real-life) psychological scores, enabling flexible and precise control over the desired level. For mental health variables, PsychAdapter was particularly effective in generating distinct text for all levels of depression and low levels of life satisfaction, while for high levels of life satisfaction, the text was less distinguishable. This evaluation demonstrates PsychAdapter’s ability to produce text that reflects precisely specified psychological variables.

### Generalization across text domains: Twitter/X vs. blog posts

By training the PsychAdapter model on diverse text domains, we can observe the representative expressions of personality across domains. Table 4 presents text generated for Twitter/X and blog users with different levels of extraversion and conscientiousness. In general, individuals tend to produce shorter texts on Twitter and longer texts on blogs. Text domain interacts with personality: low agreeableness results in more expressive and coarse language on Twitter, whereas blog authors maintain a more polite tone, even if they are disagreeable. For high conscientiousness, both Twitter and blog authors mention work and responsibilities. However, Twitter users also frequently reference working out and gym-related content, which is absent in the blog group. In general, such PsychAdapter text generation can foreground how personality and traits are reflected in different domains of text.

**Table 4:** Text generated by PsychAdapter (based on Gemma-2B) trained on tweets and blogs, for selected Big Five personalities

Dim.	Tweets	Blogs
**High** **(+3)** **Extraversion**	• i’m so ready to see my girl • i wanna see some of these girls at the mall. • i love my boyfriend. <3 • i wanna go to a party tonight but i’m gonna be alone	• i’m a little drunk.but it’s ok.it was fun.i went to the party at the house and then i went to the party at the bar.and then i got drunk. • i think i have the best boyfriend in the whole world.i mean, he’s so sweet.he loves me so much, and he loves my friends.i love him so much, and i love his friends too. • hey guys, just got back from the party.it was awesome.i think i was the best dressed girl there, but that’s not really important.i had a blast and i’m so glad i was able to go. • i’m going to be a bridesmaid for my cousin’s wedding.i’m so excited.the wedding is going to be at the red lion in chicago.i can’t wait!i’m going to be in a white strapless dress with a big bow in the back.
**Low** **(-3)** **Agreeableness**	• damn this n**** really got a big ass dick. lol. • i hate when people call me a b****. • i hate when people ask me questions i’m not ready to answer. • f*** that s*** i just want money	• i hate the people who say that you can’t have a good time without alcohol.it just doesn’t make sense.i don’t think that people are stupid, but it is just that they are so stupid. • i hate to do it, but i have to ask.do you all hate me?i know, it’s a stupid question, but i’m just curious.i know that people have different tastes in music, but do you hate me for some reason? • i am not a racist. i don’t have a problem with people of different cultures, races, religions, or sexual orientation. i do not have a problem with people of different political beliefs. • “you have to be a little crazy to be in this line of work.if you’re not, you should go back to your own damn office."i was talking to this guy who works at the same company as me and he was saying that he had to be a little crazy to work in this field.

### Generalization across language models: Gemma, GPT2 and LLaMA3

Our approach is, in principle, applicable to all transformer-based large language models. We tested the PsychAdapter architecture on GPT-2 Large^2^ (774M parameters) and LLaMA3^38^ (8B parameters). This adapter adds only a small number of parameters to the models. For GPT-2 Large, it adds 552,960 parameters (0.07%). For LLaMA3, it adds 393,216 parameters (0.004% of total parameters). The small number of added transformation matrix parameters, along with the LoRA fine-tuning mechanism, ensures that the PsychAdapters for Gemma, GPT-2 and LLaMA3 are lightweight and can be easily distributed for use alongside the base language models.

We replicated the evaluation with three levels of Big Five personality (Low, Neutral, and High) and then annotated the generated text with Claude 3.5 Sonnet. Specifically, we had each model generate 10 samples for each position (Low, Neutral, High). Our results show that, for the Big Five personalities, these models achieve similar performance to the Gemma-2B-based PsychAdapter: for GPT-2 Large, output annotations matched intended levels with 98.7% accuracy without a prompt and with 89.3% with the prompt “I like to”; for LLaMA3-8B the accuracies were 97.3% and 92.0%, respectively. This compares to 98.7% and 88.0%, respectively, for Gemma-2B in the same experiment setup.

We replicated prior experiments with five fine-grained levels with GPT-2 and LLaMA3-based using Claude as the annotator, see Fig. 4 showing similar performance to prior results on Gemma. Across five levels, for LLaMA-3, the accuracy was 67.6% accuracy without a prompt and with 71.6% with the prompt “I like to”. For GPT-2, the accuracy was 74.4% accuracy without a prompt and with 73.2% with the prompt “I like to". This also compares to 73.0% and 77.0%, respectively, for Gemma in the same experiment setup.

## Discussion

We developed a language model adapter that generates text corresponding to a profile of psychological and demographic trait scores. We trained this adapter to produce language for Big Five personality, depression, life satisfaction, and age – but in principle, any variable reflecting between-person differences can be used to tailor language generation. Our results suggest that PsychAdapter can reflect psychological traits at fine-grained levels, as evidenced by expert evaluations as well as automatic annotations by Claude Sonnet. Furthermore, we found that it produces language representing profiles that combine multiple traits in a manner that aligns with psychological theory (such as language expressing warmth and dominance, as situated in the Interpersonal Circumplex^34^). We found that this approach generalizes across text domains and language models. This work enables a deeper exploration of individual characteristics and how they are reflected in natural language, and points to a novel mechanism for modifying transformers with desired human-like traits without relying on prompting or consuming part of the context window. Furthermore, experiments demonstrated that PsychAdapter combines well with prompting to elicit trait-congruent generation for specific life domains (e.g., “My hobbies are...”).

Our research builds on prior work in text generation that aims to reflect speakers’ psychological traits. Early studies used content and sentence planning to generate personality-aligned reviews and dialogue, but were limited to discrete traits like extraversion and agreeableness^21–23^. Other approaches focused on building personalized dialogue models conditioned on attributes such as age or gender^24–26^, or a specific character persona^39^ instead of personalities backbone. Some methods integrated personality vectors into LSTM-based architectures, which are less efficient than transformers due to sequential computation^40–42^. Recent transformer-based methods use prompt engineering to insert personality-related keywords, relying on predefined heuristics^27–30^, which limits flexibility and granularity. In contrast, our approach directly incorporates continuous psychological scores into the transformer architecture, enabling fine-grained control over text generation across a broader spectrum of traits. This method simulates diverse and nuanced personality profiles, overcoming the limitations of discrete prompting methods.

Distinct from other persona- and psychological-conditioning techniques, PsychAdapter offers fine-grained control over the underlying constructs as continuous (dimensional) psychological factors rather than discrete multinomial attributes. Other works^43–45^ control generated text characteristics using categorical descriptions captured by tokens (e.g., “Female”/“Male”, “Reviews”/“Relationships”) as the context. These methods thus lack the ability to control traits dimensionally as continuous variables (e.g., placing text generation on an extraversion or gender spectrum). Many approaches^28,29,46^ leverage the zero-shot generation capabilities of frontier models to generate desired text solely through prompt engineering. In an ablation study (Supplemental Information, Section S4), we found that smaller models (e.g., those with around 2 billion parameters) showed significantly poorer performance with prompt engineering methods compared to PsychAdapter in generating text that accurately reflects fine-grained levels of personality constructs (e.g., accuracy of 0.53 for prompting versus 0.77 for PsychAdapter using Gemma-2B).

Because PsychAdapter can generate text aligned with detailed psychological profiles, it supports a wide range of potential applications. Firstly, PsychAdapter can be used to create content that matches the psychological profile of the intended audience, such as that of an interaction partner. Such applications include chatbots that express personality profiles similar to those of the users they converse with. Previous work has shown that a good fit between users and automated agents can improve the perceived quality of the interaction experience^21–23,47–51^. Similarly, content creation and summarization applications can benefit from the PsychAdapter approach to tailoring based on the demographic (age, gender) and personality composition of the audience. For example, prior studies^52–54^ have demonstrated that generative language models can personalize content (such as news articles) in ways that reflect readers’ age and interests. Further, PsychAdapter can be the basis for creating chatbots and LLM agents that simulate realistic distributions of demographic and personality scores based on empirical results of populations surveys, enabling possible “digital cohorts” of LLMs with a desired distribution of personality characteristics to estimate responses to psychological experiments^55^. For example, it is known that Liberal voters tend to have higher openness to experience^56^, and it may be desirable to vary openness when simulating different voter populations. Secondly, PsychAdapter can offer researchers a tool to explore how psychological profiles are reflected in a given domain or topic by prompting the generator (e.g., by using phrases like “I like to” or “my hobbies are”). Prior works have used natural language processing and hypothesis testing to explore the relationships between personality dimensions, mental health, and human language using regression frameworks^13,14,17–20^. These approaches, yielding results about individual words or short phrases, provide abstract, decontextualized displays of language most associated with psychological dimensions. Although such differential language findings can provide an overview of the language associated with a construct, the sparse and decontextualized representation does not capture the rich contextual information found in fully formed text generated by such tools like PsychAdapter. Furthermore, PsychAdapter provides the advantage of generating text samples across the full range of possible underlying personality scores, with direct experimental control over the underlying personality profiles and target domains through context. Lastly, although this work demonstrates the capability of PsychAdapter within the psychology domain, the approach can be extended to generate text reflecting author characteristics that can be captured with continuous scores. For example, PsychAdapter could modify text to reflect stylistic features (e.g.,degree of formality or politeness levels), tonal attributes (e.g., assertiveness or emotional intensity), and content characteristics (e.g., abstractness or novelty). PsychAdapter thus provides a general framework for modifying generated text based on continuous input variables.

At the same time, incorporating psychological traits into generative models also raises important ethical considerations. Large language models can automate and augment human language intelligence and are transforming many aspects of society. Their full impact on labor markets, online dynamics, and other downstream technologies are just beginning to be understood. This includes potential negative uses, such as their potential to generate misinformation at scale^5^. In the work presented here, we modify these models to plausibly represent more diversity among author traits. In principle, this enables more competent human augmentation (e.g., through automated training), more personalized digital experiences, and computational interlocutors that interface more seamlessly with the user. On the other hand, this technology may also aid negative or insidious applications. It is important that all content catered to individuals or given identities be marked as such. For example, as misinformation and influence operations hinge on triggering in- and out-group identities, trait-conditioned language models may imbue generated text with subtle markers of in or out-group membership and identity that may plausibly mislead the reader either to persuade or agitate.

This work also has several limitations. In this work, we have prioritized establishing the generalizability of PsychAdapter across different LLMs (e.g., Gemma, LLaMA, GPT), psychological constructs (e.g., personality traits, mental health variables), and text domains (e.g., social media, blogs). However, we leave evaluation on larger model sizes (e.g., 70B+ parameters) and larger datasets for future work, which would require substantial computational resources (LoRA could help mitigate memory demands). Additionally, for multi-trait combinations, our work focuses on illustrative examples to demonstrate the generative capability of PsychAdapter. Given the exponential complexity evaluating the interaction of personality traits and demographics is a challenging task even for expert human reviewers; Further evaluation of PsychAdapter’s multi-trait ability should be explored in dedicated further work. Lastly, while our use of a frontier LLM allowed us to scale the evaluation, it also has limitations, as training biases may overlap. In addition to the quantitative comparison with human experts, we conducted a qualitative error analysis to identify possible failure modes. Specifically, within the expert-judged data, we examined all cases where the human raters agreed with each other but disagreed with Claude. Some clear patterns emerged in Claude’s behavior. For instance, Claude appeared to fail to attend to a preference for solitary activities as a marker of introversion.

Taken together, these results demonstrate the broader significance of PsychAdapter. PsychAdapter is a novel, adaptable method and codebase for incorporating psychological traits and demographic factors into transformer-based language models, allowing fine-grained control over generated text. This approach extends the capabilities of language generation by simulating language generated across personality, mental health, and demographic profiles without relying on discrete prompt tokens. Our results show that PsychAdapter accurately generates the targeted psychological profiles, as verified by expert and automated evaluations. This development has broad implications, from enhancing AI-human interaction with trait-congruent agents to providing valuable tools for psychological research through language-based insights.

## Methods

### Dataset and Models

To train PsychAdapters, we utilize a pre-trained language-based assessment model tailored to the outcome of interest (e.g., Big Five personality traits, mental health scores, or age) along with a text corpus. The assessment model assigns “estimated” psychological scores to the text corpus at the message level. These assigned psychological scores, along with their corresponding text, serve as input-output pairs for training PsychAdapters.

For this study, the text corpus comprises two primary datasets: a collection of open-source blog posts and a set of tweets. The blog dataset^57^ comprises contributions from 19,320 blog authors aggregated from blogger.com in August 2004. The tweets dataset^58^ contains county-level language features extracted from a large U.S. county-mapped Twitter corpus. This corpus includes 1 billion anonymized tweets, collected from a random 10% sample of the entire Twitter stream (“GardenHose”) from July 2009 to April 2014. These datasets consist of voluntarily shared status updates, which provide a rich source of text data for our model. From the tweet dataset, we randomly selected 500,000 posts. For the blog dataset, we included all available 681,288 posts. Data preprocessing involved removing entries with fewer than five words and those containing links or other extraneous content, such as emoji codes and hashtags. For the blog dataset, due to the high average length of approximately 207 words per post, we only used the first 30 words of each post. We parsed each blog post into sentences and used only the first few sentences, ensuring they totaled slightly more than 30 words. The blog authors comprise 42.65% in the age range 13-17, 41.85% in the age range 23-27, and 15.49% in the age range 33–47. Each age group has an equal number of male and female participants. The median and average length of each blog post after processing are 32.0 and 36.35 words, respectively. The anonymized tweets dataset does not include any participant information. The median and average length of each tweet are 12.0 and 14.59 words, respectively.

To create the training dataset for PsychAdapter, we employed language assessment models capable of predicting psychological variables such as personality, depression, and life satisfaction from text. These models assigned estimated psychological scores to each blog and tweet message. Detailed methodologies for score assignment are provided in the following subsection. For predicting Big Five personality traits, we utilized a model built with the proposed approach in previous work^13^, which leverages topic features to predict personality scores, training using our internally collected data with participants’ consent. For the depression and life satisfaction variables, we used the models proposed in previous works^15,59^. We used the versions of these models that take in extracted topic features, as described in^13^, created by running latent Dirichlet allocation (LDA) using 2000 Facebook topics released by prior research^13^, as input for prediction. For age prediction, we employed the model proposed in previous work^33^, which uses text lexicon features as input for prediction.

For the base language model upon which PsychAdapter is augmented, we used Google’s open-source model Gemma-2B^60^, distributed at https://huggingface.co/google/gemma-2b. We also tested our method with LLaMA3-8B^38^, distributed at https://huggingface.co/meta-llama/Meta-Llama-3-8B, and GPT-2 Large^2^, distributed at https://huggingface.co/openai-community/gpt2-large.

All experiments that produced the data were approved by the university’s IRB and comply with all relevant ethical regulations. Informed consent was obtained from all participants conducting surveys.

### Methodology

**Overview:** We propose a method to modify a standard auto-regressive transformer-based language model (e.g., as used by GPT, Gemma, LLaMA) to distinguish language characteristic of given dichotomous or continuous-value human factor/trait scores. Conditioning a generative language model on continuous variables (e.g., personality scores) presents two challenges: (1) the hidden state vectors within the transformer models have orders of magnitude more dimensions (e.g., 2048 dimensions in each of the 18 layers of the Gemma-2B model) than the human factors (e.g., only 5 dimensions in the Big Five personality traits); and (2) by default, language models tend to produce the most typical language, which is often non-insightful, rather than the language most distinctive of specific traits. To address the first challenge, we introduce high-dimensional projection matrices to expand the input of human trait vectors to match the size of the transformer network’s hidden state representations. As illustrated in Fig. 1A, a separate projection matrix was learned for each layer (except the last, as it would have no influence), allowing alignment between human factors and the hidden states. The following section shows formulas in details of how we modify the standard Transformers architecture to be able to take a vector as input condition. For the second challenge, to bring out language most distinguishing Big Five traits, we used an objective that takes a lexical-based “estimated” Big Five vector (described below) as input. The “estimated” Big Five score is obtained using a lexical-based model^18^ as training data. The model was then configured to regenerate the original post from this input vector. Details of how we obtain estimated psychological scores for each message is explained in the section below.

Our model is then trained using the text reconstruction task, as illustrated in Fig. 1B, which is similar to how most language models are trained. After training, we use the model to generate text conditioned on any input vector of Big Five scores. To generate text focusing on one personality dimension, we can configure the input vector to have extreme values (at + / − *k* times of the standard deviation value) on that specific dimension while keeping the mean value for the other dimensions:

3 $$({\mu }_{1},\ldots ,{\mu }_{i}+k\cdot {\sigma }_{i},\ldots ,{\mu }_{5})$$

Here, *μ*_*i*_ corresponds to the mean, and *σ*_*i*_ corresponds to the standard deviation for a specific trait *i*. Integers *k* from the range { − 3, − 2, − 1, 0, 1, 2, 3} can be interpreted similarly to a Likert scale from 1 to 7. Combinations of input Big Five variables values will produce the corresponding profiles.

**Modifying transformer language models to generate text conditioned on psychological inputs:** Our goal was to create a language model that generates text conditioned on the Big Five personality vector. More specifically, we trained this model using a text reconstruction task (also known as autoregressive language modeling), where the input is the personality score vector associated with the text to be reconstructed. With the personality score vector fixed, the autoregressive task trains the model to generate a social media post from start to finish.

Traditional autoregressive language modeling estimates the probability of a sequence *s*_*i*_ (i.e. a social media post): $p({s}_{i})={\prod }_{j=1}^{{n}_{i}}p({w}_{j}| {w}_{1},{w}_{2},...,{w}_{j-1})$ with $({w}_{1},{w}_{2},...,{w}_{{n}_{i}})$ are the words making up the post *s*_*i*_ which has the length of *n*_*i*_. In comparison, our approach incorporates an additional personality or psychological trait vector, *ψ*_*i*_, for each input sequence. More specifically, from a set of *n* training samples {(*s*_1_, *ψ*_1_), (*s*_2_, *ψ*_2_), (*s*_3_, *ψ*_3_), . . . , (*s*_*n*_, *ψ*_*n*_)}, we seek to train the model to maximize the probability of the sequence given the psychological traits:

4 $$p({s}_{i}| {\psi }_{i})=\mathop{\prod }\limits_{j=1}^{{n}_{i}}p({w}_{j}| {w}_{1},{w}_{2},\ldots ,{w}_{j-1},{\psi }_{i})$$

Figure 1A illustrates the training process of our model for a single sample. Once PsychAdapter is trained for a given personality vector, the model can be used to generate text for any values of the input vector – for example, by sampling from high or low values of each dimension.

There is one main challenge regarding the model’s architecture in the task described above. Standard transformer-based language models, such as GPT^2^, Gemma^60^, and LLaMA^38^, frequently generate responses conditioned on text-based prompts. However, they cannot directly condition on a vector in an arbitrary continuous space, as is the case with our Big Five vectors or mental health variables. Most previous works present the conditioning as prompt text or special beginning tokens/phrases^61–65^. These approaches limit the conditioning to discrete variables and lexical features (e.g., categories of emotion words or topics) rather than continuous variables that describe the degree of a personality trait.

To condition the model on a personality vector (e.g., five personality scores), we introduced a simple modification to the standard autoregressive transformer architecture^1,2^. We build on works that inject conditional information by including it as a special first token or keyword in the sequence^44,66^. However, we modified this approach for our use case because (1) we are conditioning on continuous rather than categorical (or multinomial) variables, and (2) there is a size discrepancy between the personality vectors – typically ranging from 1 to tens of dimensions – and the hidden state representation vectors in autoregressive language models, which typically range from hundreds to thousands of dimensions (e.g., 768, 1024, or 2048 dimensions).

We addressed these challenges by using a dimensional expansion, a trainable weight matrix to linearly project the small number of continuous values (i.e. personality scores) into the larger hidden state vector. This projection is fed into the language model as the hidden state of the first dummy token at all layers, except the last layer. The model learns a different transformation matrix for each layer since the layers are known to vary in the type of information they encode from more syntactic to more semantic^67^.

That is, at each layer *l*, we add to the model a transformation matrix ${W}_{l}^{trans}$ (e.g., size 5 × 2048, with 5 corresponds to Big Five personality scores input size, and 2048 corresponds to the hidden state representation size of Gemma 2B) to transform the input psychology scores vector *p* (e.g., size 1 × 5) to the hidden state vector *h*_*l*_ (e.g., size 1 × 2048): ${h}_{l}=p\times {W}_{l}^{trans}$. The expanded personality vector is thus able to influence layers after the input layer. Figure 1A illustrates the architecture of our modified generative model.

Particularly, each transformation matrix will have the shape of:

5 $$[{\mathrm{latent}}\_{\mathrm{size}},\,{\mathrm{num}}\_{\mathrm{key}}\_{\mathrm{value}}\_{\mathrm{heads}}\cdot {\mathrm{head}}\_{\mathrm{dim}}]$$

Where *latent*_*size* is the size of input variables (e.g., 5 for Big Five personalities, 1 for depression/life-satisfaction, 2 for combination of depression/life-satisfaction and age), *num*_*key*_*value*_*heads* is the number of the transformer block’s key and value heads, *head*_*dim* is the dimension of the each head. For each layer, we have one transformation matrix correspond to the hidden states’ key and one transformation matrix correspond to the hidden states’ value. We have in total 2 × (*num*_*hidden*_*layers* − 1) of transformation matrix, as the last layer does not require.

This modification of the language model adds only a smaller number of parameters to the model. For Gemma 2B, it adds 55,296 parameters (0.002% of total parameters). For GPT-2, it adds 552,960 parameters (0.071%). For LLaMA3, it adds 393,216 parameters (0.005% of total parameters).

For the base language model, we used LoRA^68^ PEFT method to expedite the finetuning process and get the model running on limited GPU memory. The LoRA configuration is set to: *r* = 8, *alpha* = 32, *lora*_*dropout* = 0.1, and *target*_*modules*=[*”q*_*proj”*, *”o*_*proj”*, *”k*_*proj”*, *”v*_*proj”*, *”gate*_*proj”*, *”up*_*proj”*, *”down*_*proj”*]. With this setup, the total number of trainable parameters, comprising the base language model’s LoRA weights and transformation matrix weights, for Gemma-2B model is 0.39% of total parameters. For GPT-2 Large and LLaMA3-8B, the total number of trainable parameters is 0.33% and 0.26% respectively. We trained the model on the training partition of blogs and tweets, with a batch size of 64, using constant learning rate of 5e − 5, using NVIDIA RTX A6000 GPUs.

After training, thanks to the small number of PsychAdapters’ trainable parameters (LoRA weights and transformation matrix weights), they can be easily distributed to be used with the base language models. For the same base language model, different PsychAdapters (e.g., adapter for Big Five, adapter for life-satisfaction, adapter for depression) can be used by plug-and-playing the adapters to the model. Note that while in this work, we tested our methods with Gemma, GPT-2, and LLaMA models, our proposed modification can be used for most modern text generative models built upon the transformer^1^ building block, and hence, can be modified in a straightforward manner with our proposed approach.

**Obtaining estimated psychological for messages:** To train the model, as illustrated in Fig. 1, each training sample includes a text message and its associated Big Five personality scores. However, Big Five personality scores are typically considered at the participant level, rather than the message level. Therefore, we propose a method to estimate personality scores at the message level using a predictive model trained at the participant level, which predicts participants’ personality scores based on their authored text. Specifically, we built internally a participant-level model based on the method proposed in the previous study^13^, which uses machine learning methods to infer human psychology from social media footprints. Our model is trained on a combination of social media text and corresponding personality scores collected from the works^69–71^, to predict the Big Five personality traits of an author based on their text collection. The input to this model consists of the 2000 LDA Facebook topic features^13^ extracted from a participant’s text collection. We applied this participant-level model to message-level samples, with the extracted 2000 Facebook topic features as input, to produce an “estimated” personality score for each message. This model can then annotate each social media post with the corresponding personality scores, effectively functioning as a “teacher” for the generative model to learn from. One advantage of this approach is that, after training the participant-level predictive model, we can generate as many training samples as needed for PsychAdapter, leveraging the abundance of available social media posts.

To formulate the pipeline, we denote the psychological score of one participant *p* as ${\psi }_{p}^{(part)}=({\psi }_{p,1}^{(part)},{\psi }_{p,2}^{(part)},...,{\psi }_{p,t}^{(part)})$, where *t* is the number of psychological scores. We denote ${X}_{p}^{(part)}$ as the vector of the participant’s words frequencies while ${X}_{m}^{(mess)}$ denotes a vector of word frequencies from message *m*, with the same size and order as ${X}_{p}^{(part)}$. The participant-level model (i.e., model in previous work^13^) can be formulated as *t* matrices (*W*_1_, *W*_2_. . . , *W*_*t*_) that aims to approximate the participant’s psychological scores from their words frequencies vector ${X}_{p}^{(part)}$: $({W}_{1}\times {X}_{p}^{(part)},{W}_{2}\times {X}_{p}^{(part)},...,{W}_{t}\times {X}_{p}^{(part)})$. These *t* matrices are learned from minimizing the mean squared error of input features ${X}_{p}^{(part)}$ and output $({\psi }_{p,1}^{(part)},{\psi }_{p,2}^{(part)},...,{\psi }_{p,t}^{(part)})$ across the set of all participants *P*:

6 $$\begin{array}{rcl}{W}_{1} & = & \arg \mathop{\min }\limits_{p\in P}{\left\Vert {\psi }_{p,1}^{(part)}-{W}_{1}\cdot {X}_{p}^{(part)}\right\Vert }_{2}^{2}\\ {W}_{2} & = & \arg \mathop{\min }\limits_{p\in P}{\left\Vert {\psi }_{p,2}^{(part)}-{W}_{2}\cdot {X}_{p}^{(part)}\right\Vert }_{2}^{2}\\ & \vdots & \\ {W}_{t} & = & \arg \mathop{\min }\limits_{p\in P}{\left\Vert {\psi }_{p,t}^{(part)}-{W}_{t}\cdot {X}_{p}^{(part)}\right\Vert }_{2}^{2}\end{array}$$

After learning the participant-level psychological scores predictive model as the *t* matrices (*W*_1_, *W*_2_, . . . , *W*_*t*_), we apply this model to the message-level. For each message, we use the learned participant-level model to produce the estimated psychological scores per message, ${\psi }_{m}^{(mess)}$ by applying the matrices to the message’s words frequency vector ${X}_{m}^{(mess)}$:

7 $$({\psi }_{m,1},\,{\psi }_{m,2},\,\ldots ,\,{\psi }_{m,t})=({W}_{1}\cdot {X}_{m}^{(mess)},\,{W}_{2}\cdot {X}_{m}^{(mess)},\,\ldots ,\,{W}_{t}\cdot {X}_{m}^{(mess)})$$

The estimated psychological scores for all messages were then used as the conditioning vector for training the language model of PsychAdapter as described above.

## Supplementary information

Supplementary materials

## Data Availability

The processed data used to create PsychAdapter, along with the model itself, are open-sourced for research purposes. Human evaluations data are included in the section S2 of Supplementary Information.

## Code availability

All the scripts used for modeling PsychAdapter, as well as those for running training and inference, can be found at https://github.com/humanlab/psychadapterand are open-sourced for research purposes.
