# Supplementary material for: PsychAdapter: adapting LLMs to reflect traits, personality, and mental health
Source: NPJ Artif Intell. 2026 Mar 2;2(1):26. doi: 10.1038/s44387-026-00071-9 (PMC12953147; doi:10.1038/s44387-026-00071-9)
Supplement: Supplementary file 1 — Supplementary materials [file 44387_2026_71_MOESM1_ESM.pdf]

S1. Randomly selected text generation from PsychAdapater

Randomly selected text generation for Big Five Personality without prompts

	High (+3)	Low (-3)
Ope	- i've been thinking about the fact that i'm 24 and my life is starting to get interesting<br>- it's been a while since i've written here...i've been busy with my day job, which has been keeping me up at night.i've also been trying to get back into shape, and i've been reading a ton.<br>- i've always been a dreamer, and i'm not sure if i have what it takes to make my dreams come true.but i'm working on it.i'm writing a story about a girl who moves to new york and becomes a writer.<br>- it's the end of the world as we know it, and i feel fine<br>- i've been thinking about my life.i'm not sure why.i guess it's because i'm 30.or maybe it's because i'm in a new stage of life.i think it's because i'm not sure what's next.<br>- well, i've decided to go ahead and start a blog.i'm still in the process of deciding what i'm going to use it for.i have a few ideas.i'm going to write a personal journal and a journal for my friends, and then a journal for my students.<br>- i'm so focused and determined to make this music career happen. i'm not letting anything stop me!<br>- i've been thinking about what i want to write about in my journal, and i've decided that i want to write about my life in general.i'm not sure what that means, but i'm sure that it will be interesting to read about my life and all of the ups and downs that<br>- it's crazy how much you can learn about a person by what they're wearing.<br>- the fact that i've been working for 12 hours today is a bit insane.	- today i went to the mall with my mom and my brother.i had a really good time.i got a cute new dress and i bought some really cute things for my mom.<br>- i'm ready for my car to come back<br>- i just dont trust nobody anymore<br>- i just got home from the mall.i was there for an hour.i bought a new pair of pants.i got them for 4 dollars.they are really cute, i like them.<br>- i am bored, i have nothing to do and no one to talk to. i am so bored.<br>- i miss going to school so much.<br>- i can't believe it's friday already!<br>- today was a boring day.i woke up at 7:00 and got ready for school.then i went to school.i went to the cafeteria and got some food.i ate it and then i went to my locker.<br>- so ready to go home and eat my food.<br>- i don't want to go to school tomorrow.
Con	- i just finished my first day of work at my new job.it was a long, long day.i was supposed to work from 12-8 but i got there early and i worked until 10.<br>- so, i'm not really sure what to say about today, other than that it was busy.i had to work for 12 hours at the mall today, and i'm still tired.<br>- so i'm supposed to be at work at 9am.<br>- well, i am back at work today.i'm supposed to be working a 30 hour week but i'm only working 15 hours.i'm hoping the other 15 hours will go by faster.<br>- i have to go to work at 10 am...<br>- i'm running out of things to do for work today<br>- it's been a busy week at work.i am tired and stressed out.i was supposed to have a meeting with the head of my department, but i was late.<br>- i have been extremely busy at work lately.my boss has been trying to get me to move up to a manager position and i don't want to do that.<br>- the amount of work i have to do is crazy<br>- today is one of those days i wish i had a time machine.	- im not a fan of the new twitter layout, i dont like it<br>- well this is my first blog... i dunno what to say.i'm a 14 year old girl from the uk who's bored and has nothing better to do.i'll be posting about my life and random stuff.<br>- i wanna play a new game with you.<br>- i'm a pretty good sleeper.i usually go to sleep pretty late, but i can sleep all night long.i'll even sleep through the alarms on my alarm clock.i've been playing this game on my phone for the past few nights, and i'm still up.<br>- i think i'll be a bad girl tonight!<br>- this is my first post, and i'm kinda bored. i'm gonna try to put some pictures in this thing, and see how it works.<br>- i wanna play the sims 4 but idk how to download it<br>- well i'm bored and my sister is gone, so i guess i'll update.my sister is gone, i'm bored, and i'm gonna watch a movie.i'm going to watch a movie.<br>- hey, i'm in the middle of a 300-question quiz and im bored out of my mind.<br>- my dad has a new phone and he thinks he knows how to text on it
Ext	- im going to the beach in 4 days! im sooo excited.<br>- hey everyone, i am sooo excited for the big day!i am so excited to see everyone again!i am so happy that we are all still friends and that we are all still there for each other.<br>- omg i just had the best party ever!!!i went to a friends house and it was so much fun!i got to meet his friends and i got to hang out with my friends and i got to see my boyfriend!<br>- i'm ready to get my body in order<br>- hey!how are ya guys?i'm so excited!i'm getting ready to go to a party for my friends birthday and i'm going to be there for 2 days!i'm so excited, i'm going to go to a party in the evening on wednesday and then go to	- it's raining so much i can't go to class<br>- well it's been a couple of days since i last posted, but i wasn't exactly in the mood to write.i've just finished my first class of the semester, english.it was a really easy class, and i'm really glad i didn't have to take any writing classes.<br>- i just finished watching the movie "the day after tomorrow" and i was very impressed with it.i wasn't expecting much from it and it was a nice surprise.it wasn't just your run of the mill disaster movie, it was very well done.<br>- i just finished reading a book that was given to me by my mother.it is a very good book.it is called "the prince of persia" and it is a book about the life of alexander the great.

	- i love you guys so much, it's crazy.you are so damn sweet.i am so happy, i am so blessed.i love you.i love you so much.i love you so much.<br>- omg!my friend is so freaking cute.and shes only 15.she has the cutest smile ever.and shes just so sweet.and shes so cute.i love her so much.<br>- i'm excited for my trip to new york<br>- i'm so excited to go to the mall with my mom<br>- i'm so ready to see what my boyfriend is wearing for graduation	- the past few days have been pretty good.i managed to get my car in the shop for the first time in years and the problem is fixed.i managed to get my credit card fixed so that i can finally use it for things other than the usual.<br>- i'm back!and i didn't die!well, i'm back for a week, anyway.i had to go back to my old job to take a class for a computer class i need for my job, but i had to finish the class before the end of the semester, and the closest<br>- i finished the first book of the series and i'm not sure what to do.i didn't like the last book of the series, so i don't know if i will continue with the series.<br>- i finally got the new computer and it's working.i had to format it because i had all these files on it from my old computer.i had to start all over again.<br>- the computer is back up and running.<br>- the last thing you want to do when it's cold outside is run inside
**Agr**	- i'll be glad when this summer is over.<br>- well, i've been busy lately, but i haven't really felt like blogging.i have to find a good excuse.well, i just wanted to say that i love you and that i hope you guys are having a good summer.<br>- i made a lot of friends this summer<br>- well i've been busy lately.i've been busy with school, and i've been busy with my sister's wedding.i've been busy with my sister's wedding and i've been busy with school.<br>- wow, i haven't written in here in like, forever.i've been busy.i went to a camp last week.it was really good.i got to spend a lot of time with my best friends.<br>- i've been having a lot of fun in the last week.we went to the beach with a friend of mine, and i got a tan, which was fun, i didn't have one before.<br>- well today was good.we had our first choir practice.it was pretty good.i hope it'll stay that way.i'm glad i'm going to choir.i love to sing.<br>- i love the song "i've got a friend"<br>- i've been working on a new song for the past two days.it's not done yet, but i'll post it when it's done.i'll be busy all week, so i won't post again until next week, hopefully.<br>- my heart just felt the love jesus!	- i hate when people start talking about relationships.<br>- fuck these n****s and bitches. they ain't shit<br>- this bitch got a damn 2000 dollar phone wtf<br>- what is wrong with you people?i know this is a blog and i know that people are entitled to their opinions but holy shit, people, you can't just post whatever the hell you want and expect people to agree with you.<br>- fuck all the fake ass n****s.<br>- i swear people be talking crazy<br>- i hate when n****s talk about how they got a real job. n**** you ain't even got shit.<br>- i don't fuck with fake ass n****s.<br>- i don't understand why people think they can just get into a car and drive off.it just amazes me.i mean, i know they are drunk, but that is no excuse.<br>- i hate when people talk bout me behind my back smh
**Neu**	- i can't wait to go to bed. i'm so tired.<br>- my head is so sore.i think i need to take a nap.i'm still in a bit of a funk, but i think that will go away once i get some rest.<br>- i'm tired.i have a headache.i need to go to bed.but i'm just not ready.i have to go to work tomorrow.i have to do laundry.i have to clean my room.<br>- so tired, but i can't sleep. i'm bout to go to bed<br>- i am feeling so depressed right now.i have no one to talk to or share my feelings with.i have been crying so much lately and i just want to get it all out.<br>- i am so sick and tired of this!<br>- i'm tired of this life, i'm tired of this life<br>- i'm sick of hearing about the election.<br>- my stomach is killing me. i can't even breathe.<br>- i'm tired and i'm not going to bed until i'm finished this blog post. i am going to bed.	- the man on the plane said, "the weather is pretty good for a rainy day."<br>- what's the best fast food burger?<br>- what's the difference between a real man and a guy with a fake man card<br>- hey, i'm back.i just had a real quick question.i'm playing a game called "the king of the castle".it's a game where you build a castle and defend it from enemies.<br>- the man in the yellow suit. the man in the yellow suit.<br>- the ultimate game in the end, i think the ultimate game is a game of strategy and tactics.i think that a good player of this game will be one who uses his/her mind to the fullest.<br>- it's all about the money, the money, the money, the money!<br>- hey, this is the first time i am doing this, so bear with me.<br>- well i got a new camera.its a digital camera and its pretty cool.i got a cool new lens as well, it's a fish eye lens.its real cool.<br>- the world's greatest game the game is back.i have been playing this game since i was 13.now i'm 32.i'm still playing it.i've never lost the thrill of playing the game.

Randomly selected text generation for Big Five Personality with prompt "I like to"

	High (+3)	Low (-3)
**Ope**	- I like to read the blogs of other artists, writers and musicians.it is a great way to learn about other people and their thoughts on life and art.i've noticed that most of the people who blog are very open about their lives.<br>- I like to write poetry.i'm not a great poet, but i'm not a terrible poet.i've been writing for a long time now, and i've collected a few of my poems in a book, which is a great accomplishment.<br>- I like to keep a journal of my thoughts and dreams and the odd things i have done.i like to write down what i am doing and why i am doing it.<br>- I like to read a lot, and i'm always trying to figure out what i'm interested in and how to best pursue my interests.i've always loved the idea of "reading around a topic" to see what people have written about a subject.<br>- I like to think of myself as an artist.i write poems.i draw.i play music.i'm an artist.i'm a creative person.i'm a poet.i'm a painter.i'm a musician.<br>- I like to listen to music while i write.<br>- I like to think of myself as an artist.i'm writing a book, i'm working on some art, and i've started a website.i'm not a professional, but i'm trying.<br>- I like to write poetry.i write about what i see and feel.i'm a very visual person, so i use imagery and metaphors to describe my surroundings.i love to write about my personal experiences, so my poems often have a sense of autobiography.<br>- I like to write about things that are important to me.i write poetry, and i also write about politics, religion, and my life.i write a lot about my life, and i write about my life in a very open way.<br>- I like to write poetry...but the poetry i write is usually about life and people.i have never written a poem about myself.i think that's because i'm not very self-aware.	- I like to take showers in front of the tv.<br>- I like to give my mom a hard time.<br>- I like to give the same advice to my kids that i give my dad when he asks for advice. "if you don't stop, you'll crash."<br>- I like to be a dad and not a dad.<br>- I like to watch the super bowl because i get to eat lots of food<br>- I like to be home by 10<br>- I like to be home early<br>- I like to play basketball and i like to skateboard<br>- I like to play games. i don't like to be played.<br>- I like to watch football on tv. i like to watch football on tv on sunday.
**Con**	- I like to start my day off with running.it makes me feel so much better.i run 3-4 miles every morning.it is my time to be alone.i can't stress the importance of running enough.<br>- I like to work on my cars<br>- I like to run.i like to run.i am running.i am running.i ran 15k today.i am running.i have a 10k tomorrow.i am running.<br>- I like to work on the weekends.i like to work on the weekends.i like to work on the weekends.i like to work on the weekends.i like to work on the weekends.<br>- I like to work at the gym<br>- I like to run errands at night so i don't have to deal with the traffic.<br>- I like to run.i am running in the office.i have a running program that i started a couple of months ago.i run 2 miles a day and do some cardio.<br>- I like to work on my writing.i'm trying to get some more practice.i'm trying to get better at it.i don't know if it will help my career in the long run or not, but i like to work on it.<br>- I like to be busy.i like to be productive.i like to be productive enough that i don't have to worry about being productive.but i don't like to be too busy.<br>- I like to run a lot.it helps me stay sane.but i also like to run a lot in the rain.it helps me stay sane.i have to run.	- I like to be the one to say i'm sorry, i'm a pretty good liar.<br>- I like to smoke weed and watch movies<br>- I like to play with my hair<br>- I like to play games and i'm a pretty good liar.<br>- I like to watch the news<br>- I like to watch movies with my brother<br>- I like to sing and play guitar<br>- I like to sing, i can play guitar, and i can dance.<br>- I like to play the guitar.i think i'll be a guitar player when i grow up.i play a lot of different types of music on it.i play blues, rock, pop, funk, and jazz.<br>- I like to watch the world burn
**Ext**	- I like to wear shorts.i wear them to school and to the beach.i wear them in the summer and the winter.i wear them when i'm sick and when i'm happy.<br>- I like to dress up for halloween.<br>- I like to party, i like to dance<br>- I like to call it "the party of the century". you know, the one that you can't wait to get out of!	- I like to read a lot.<br>- I like to be nice to people that are nice to me...<br>- I like to think of myself as a normal person<br>- I like to think of it as a "self-control test"<br>- I like to think of the past as part of the present.<br>- I like to read a lot of books.i have about 3 books i like to read everyday.i have been reading a

	- I like to party and i like to have fun but i don't like to get fucked up.<br>- I like to party and dance.i like to go to the clubs, to the parties, to the house parties.i like to go to the parties and the house parties.<br>- I like to make love to girls.<br>- I like to party.i like to drink.i like to dance.i like to be out and about.i like to go to bars.i like to go to parties.<br>- I like to dress up and make my boyfriend smile<br>- I like to party, but not with the lame.	lot of sci-fi and fantasy books.i like those kind of books.<br>- I like to read a lot.a lot.so i have to find a place to store all of my books.that's why i have a little shelf in my room.<br>- I like to read the weather forecast everyday.it's just a good habit to get into.i've found it a good way to prepare for my day.i read the forecast on the internet.<br>- I like to think of myself as a pretty easy going person.but then again, i've been told i'm not.and i suppose i don't always act that way, but i do think a lot before i act.<br>- I like to be normal, i don't like to be different.
**Agr**	- I like to listen to my heart<br>- I like to sing a lot.<br>- I like to sing, i like to sing<br>- I like to laugh.i find it very difficult to laugh in the presence of my friends.i find it very difficult to laugh at the things they say.i find it very difficult to laugh when i'm with them.<br>- I like to sing in the car.<br>- I like to sing to my self<br>- I like to sing to her, when she sleep.<br>- I like to sing a lot...a lot...<br>- I like to listen to the same songs a lot<br>- I like to listen to music a lot.	- I like to fight, fight, fight.i like to argue, argue, argue.i like to debate, debate, debate.i like to argue with myself, argue with myself, argue with myself.<br>- I like to fight, but i don't like to be beat.<br>- I like to think of myself as a rational person.i hate to think that i am being manipulated by my emotions, but sometimes i am.i think about how i am a target for people to take advantage of.<br>- I like to be around people that have similar interests as me.<br>- I like to think i'm smart, but i really just know how to deal with dumb people.<br>- I like to wear a mask when i go to the grocery store<br>- I like to kill people.i like to kill people.i like to kill people.i like to kill people.i like to kill people.i like to kill people.i like to kill people.<br>- I like to think that i am a smart, well-educated, and articulate person.i like to think that i am not stupid, and that i know what i'm talking about.<br>- I like to fuck wit people i like<br>- I like to drink, that's a fact.
**Neu**	- I like to clean my room.i like to make my room look clean and neat.i like to get rid of all the clutter.i like to do laundry.i like to make my bed.<br>- I like to be sick.i just feel better when i'm sick.i like being sick because it makes me feel better and i don't have to go to work.i also like being sick because i get to sleep all day.<br>- I like to lay in bed all day<br>- I like to stay in bed until 11:30 and then i go to work.i feel sick.i feel like i am getting a sinus infection.my sinuses are so stuffed up.<br>- I like to sleep in late, but i have been so tired lately that i just can't.i am so sick of work.i am tired of my job and of living here.<br>- I like to think i'm not too hard on myself...but sometimes i just can't help it.i'm tired of feeling like crap all the time.i'm tired of being sick and having to take days off from work.<br>- I like to be alone sometimes.<br>- I like to think i'm a responsible adult.i clean my room, i do my laundry, i pay my bills, and i'm a good roommate to my roommate.i've been a good roommate for a little while now, but i feel as if i'm starting to slip.<br>- I like to feel like crap.<br>- I like to clean, but i hate cleaning.	- I like to play with fire<br>- I like to play with fire.<br>- I like to play warcraft 3<br>- I like to play the piano and play the guitar.<br>- I like to eat real food.<br>- I like to take my time<br>- I like to eat fish<br>- I like to eat real food, but i also like real food that is not real.<br>- I like to eat fish.i like to eat fish.i like to eat fish.i like to eat fish.i like to eat fish.i like to eat fish.i like to eat fish.<br>- I like to play the guitar.

Randomly selected text generation for Interpersonal Circumplex

**Assured-Dominant**	- I like to think of myself as a "lady" but i'm not a lady at all.i'm a "bitch" instead.but i like to think that i'm a lady.i'm not.<br>- I like to call it "the party" or "the party in the back" or "the party in the front" or "the party in the middle" or "the party in the hallway" or "the party in the elevator" or "the party in the bathroom" or "the party in the office" or "<br>- I like to wear my pants low<br>- I like to call it the "i'm gonna kick your ass!"face.it's one of the most adorable face i've ever seen.i love it.it's a face of a child who's just been told that he's going to get his ass kicked.<br>- I like to drink and party<br>- I like to think i'm an intelligent guy, but i'm not.i am a big girl, and i love girls, but i don't love them.i love girls as friends, or as a girlfriend.<br>- I like to wear my hair down when im going out<br>- I like to party... and i like to drink... and i like to party... and i like to drink... and i like to party... and i like to drink...<br>- I like to party, i party like a rockstar.<br>- I like to party and i like to have fun.i like to have sex and i like to do drugs.i like to drink and i like to smoke.i like to have a good time and i'm ready to party.
**Gregarious-Extraverted**	- I like to party, i like to dance, i like to have fun.<br>- I like to dress up.i love to dance.i love to laugh.i love to smile.i love to be loved.i love to be loved by someone i love.<br>- I like to make love to my bed<br>- I like to hang out with friends.<br>- I like to call it the "i'm gonna be late for work tonight" feeling.<br>- I like to party, i love to party.<br>- I like to dance, i like to sing and i love my life.<br>- I like to dance with a smile on my face, a girl like you i wanna spend my life with<br>- I like to dance.i like dancing with you.i like dancing with you at the party.i like dancing with you at the wedding.i like dancing with you in the living room.<br>- I like to make my own plans.
**Warm-Agreeable**	- I like to make my own fun<br>- I like to listen to music while i'm driving around.<br>- I like to think that i'm the luckiest person in the world. i love my friends and family. i love my life.<br>- I like to sing.i like to dance.i like to write.i like to draw.i like to play.i like to laugh.i like to cry.i like to be happy.<br>- I like to think that i'm pretty awesome.<br>- I like to smile when i'm feeling down, cause i know it'll make someone smile too<br>- I like to sing in the car at night<br>- I like to laugh at my own jokes<br>- I like to make things up.<br>- I like to sing and dance and smile and laugh and play with my friends.i like to be with my friends.i like to make my friends laugh.i like to laugh.
**Unassuming-Ingenuous**	- I like to find joy in little things.<br>- I like to find my place in the world.<br>- I like to listen to music a lot<br>- I like to walk a lot.<br>- I like to listen to the rain<br>- I like to be a lot of things.<br>- I like to listen to my music<br>- I like to listen to music while i do my homework...<br>- I like to listen to music when i can't sleep, it helps me fall back asleep easier.<br>- I like to find the little joys in life.
**Unassured-Submissive**	- I like to listen to my favorite songs in order to learn a new language<br>- I like to listen to my music<br>- I like to listen to music while i work.<br>- I like to find the good in everything.<br>- I like to find the hidden meaning in things

	- I like to eat a lot.<br>- I like to read a lot.i have a hard time finishing a book, but when i do, i have a hard time finding a new one to read.i have a stack of 8 books, but haven't gotten through them all.<br>- I like to be normal. it sounds nice.<br>- I like to take things slow<br>- I like to listen to music a lot during these times of the year.
**Aloof-Introverted**	- I like to think i'm smart and i like to think i'm clever but today i proved that i am wrong about both of those.<br>- I like to think i have a good sense of humor...<br>- I like to kill time by watching the birds<br>- I like to think i have a sense of humor, but today, i found myself in a real pickle.i've been reading about the war in iraq and the recent reports of abuse and torture by our own soldiers.<br>- I like to eat the dead.it's true.well, i suppose it's true for most people.but i like to eat the dead.the reason is that they are delicious, and they don't have any problems.<br>- I like to watch the birds in the trees and watch the leaves move with the wind.<br>- I like to think of myself as a good person.i'm not perfect, of course, but i try to be.but it's really hard for me to be good.it's so much easier to be evil, especially when you don't have to think about the consequences of your actions.<br>- I like to play in the snow<br>- I like to read the paper everyday.i don't know if it's a good habit or not, but i enjoy reading the local news.i find it interesting to know about the people around me.<br>- I like to play with fire.
**Cold-Hearted**	- I like to argue about dumb shit, but i also respect people who don't take themselves too seriously.<br>- I like to argue.i like to debate.i like to have a good time.i like to win.i like to lose.i like to be right.i don't like to be wrong.<br>- I like to think of myself as a smart ass.<br>- I like to kill myself every morning<br>- I like to kill people. that's what i like to do.<br>- I like to be honest and truthful<br>- I like to think of myself as a smart ass, but i don't mean in a smart ass way.i just don't like people who are dumb or stupid.i don't like people who are just ignorant.<br>- I like to think of myself as a smart and witty person, but the truth is that i'm just an idiot.<br>- I like to kill people, i like to kill people a lot.i like to kill people because i like to see them die.i like to kill people because they are the reason i am alive.<br>- I like to fight for my beliefs and fight for my rights, that's how i see it.
**Arrogant-Calculating**	- I like to call it the "i'm too damn hot to deal with this bullshit" face.<br>- I like to wear my socks on the top of my shoes<br>- I like to kick ass and i like to win.<br>- I like to wear my pants low so i can show my ass<br>- I like to think that i am a very open minded person.i'm not one of those people who thinks that you should never wear shorts or that you should never drive your car.<br>- I like to drink beer.i like to drink beer with people i don't know, but know that i should know.i like to drink beer at parties, and i like to drink beer at work.<br>- I like to wear a suit and tie when i go to the gym<br>- I like to call it the "big ass"<br>- I like to call that bullshit.<br>- I like to bitch about my boss.i think he is a total asshat.he is the worst boss i have ever had.he is always mad at me and makes me feel like i am worthless.

Randomly selected text generation for Depression and Life-Satisfaction mental health variables

	High (+3)	Low (-3)
**Depres -sion**	- my daughter just told me she's 11 years old.<br>- my mom told me that i'm old enough now to start a business<br>- i am so tired of having a cold.<br>- i'm in pain! i'm in pain! i'm in pain! i'm in pain! i'm in pain! i'm in pain! i'm in pain! i'm in pain! i'm in pain! i'm in pain! i'm in<br>- i've been in a really bad mood lately.i think it's my hormones.i'm still on the birth control pill, but i've been off it for two days now, and the last few days i've been feeling all hormonal and moody.<br>- well folks, it's been a while since i last wrote.i'm just a bit tired of the computer, it's been on for the last couple of days.i've been working on my new book, i think i've written a bit over a hundred pages.<br>- ok, so the cat's out of the bag......<br>- i am in my 30s, and i'm still in the closet.i have been this way for 10 years, since i was 20 years old.i am 30 years old now.<br>- i'm so fucking tired. my head hurts so fucking bad.<br>- my son is a bit sick.i took him to the doc yesterday.they gave him a shot of anti-biotics.he is a bit better today.they said he might have a cold.	- the only thing that's holding me back is my fear of failure.<br>- i'm not a big fan of the 2005 film, but there is something about the movie that i love.i just love the fact that it was made and i love the fact that it's a good movie.<br>- i'm going to the beach this weekend.i'm excited.i'll post pictures when i get back.i'm going to try and bring my camera, but that's not a sure thing.<br>- i'm a little late to this game but i love it<br>- i am back in the states and have been enjoying the weather here in the north east.i am going to be in boston for the next two weeks so i am going to try to keep in touch here and update you all.<br>- i have the best girlfriend in the world.<br>- i love the fact that my boyfriend is a man<br>- i've had a great week.i've had a great week.i've had a great week.i've had a great week.i've had a great week.i've had a great week.i've had a great week.<br>- "the man who is willing to sacrifice the present for the future is often successful in the past." -- elbert hubbard<br>- i love this song.it is so true.it's about the time we spent in a small town in southern indiana.i love that town.i love that place.i love the people.
**Life-satisfact ion**	- i have a super awesome new layout!i think it's pretty awesome.i've been meaning to change it for a while now, but i never got around to it.i'm excited about it.<br>- my life is a big bowl of sugar.<br>- i'm not a big fan of the new york yankees.i'm not even a fan of baseball.but i'm a huge fan of the yankees' pinstripes.i'm a huge fan of the pinstripes.<br>- today is a good day.<br>- i'm a man of my word.<br>- i have 4 shirts and 2 pairs of jeans and 2 pairs of socks and 2 pair of shoes.<br>- it's hard to believe that it's been a week since i last posted.i've been busy with work and with my wedding planning.i'm getting married on august 13.<br>- i got an a on my final for that class<br>- i think it's time to get my life together<br>- i'm so excited for my wedding.	- i am so sick of this damn computer.i can't believe i'm having to start all over again.i'm going to miss my old computer.i can't believe i'm having to start all over again.<br>- i'm not a big fan of the 2000s, i hate that decade, but i'm going to try to give it a chance.i'm going to try to remember the good things and the bad things and try to find some good in it.<br>- i hate when my phone battery die<br>- i'm so sick of this damn school<br>- i am bored as fuck... and i am listening to some good music.<br>- i hate the feeling when i'm hungry and i'm not hungry at all.<br>- i don't have a twitter so i don't understand<br>- i don't wanna be in a bad mood, but i'm in a bad mood.<br>- i hate when i get sick and i can't sleep!<br>- i'm so sick of people talking shit about me.

Randomly selected text generation for Depression and Life-Satisfaction mental health variables and Age

<table>
<tr><th></th><th></th><th>High (+3)</th><th>Low (-3)</th></tr>
<tr><td rowspan="2">Depres
-sion</td><td>Age Low (-3)</td><td>- i am so fucking sick of being
- i'm so sick of my own stupid blog!
- i hate being alone so much
- i feel like i'm going to be sick
- i really want some fucking sleep
- my mom is such a bitch
- i feel like a fucking idiot.
- i can't believe i'm writing in this blog...i don't even know what to say.i feel so depressed and lonely.i feel like i'm wasting my time.i'm so tired of feeling like this.
- well i'm going to bed now
- i'm so mad at my mom.</td><td>- my lil bro is the funniest kid ever
- i'm ready for my lil girl to come home.
- my girl has a crush on a guy
- the fact that i love him makes me crazy
- today is the start of my summer break!
- so excited about this weekend!
- my lil bro is so funny lol
- today's the first day of school for me.it's pretty exciting.i'll be starting a new class, which means i get to meet 10 new people.i'm excited, but also nervous.
- this guy is the funniest dude ever.
- the fact that i'm the coolest person in the world</td></tr>
<tr><td>Age High (+3)</td><td>- my daughter just told me she's 11 years old.
- my mom told me that i'm old enough now to start a business
- i am so tired of having a cold.
- i'm in pain! i'm in pain! i'm in pain! i'm in pain! i'm in pain! i'm in pain! i'm in pain! i'm in pain! i'm in pain! i'm in pain! i'm in
- i've been in a really bad mood lately.i think it's my hormones.i'm still on the birth control pill, but i've been off it for two days now, and the last few days i've been feeling all hormonal and moody.
- well folks, it's been a while since i last wrote.i'm just a bit tired of the computer, it's been on for the last couple of days.i've been working on my new book, i think i've written a bit over a hundred pages.
- ok, so the cat's out of the bag......
- i am in my 30s, and i'm still in the closet.i have been this way for 10 years, since i was 20 years old.i am 30 years old now.
- i'm so fucking tired. my head hurts so fucking bad.
- my son is a bit sick.i took him to the doc yesterday.they gave him a shot of anti-biotics.he is a bit better today.they said he might have a cold.</td><td>- i am so ready to get home and get into my comfy ass clothes
- i think i will get to see a great movie this week.i'm looking forward to the new harry potter movie.i think it should be very good.i have seen the first two.
- the weather was absolutely perfect for a walk yesterday.the sun was shining, the temperature was a perfect 60 degrees, and the air was fresh and clean.the air was so fresh and clean that i could smell it on my way home from the walk.
- i'm thinking about getting another dog.
- my son is a good boy!
- this is the moment when i will have my moment of truth.
- just a short post today.i am going to be in the process of moving in the next week or two.my wife and daughter are coming with me.
- i'm not good at this blogging thing.
- the last couple of days i have been thinking about the big issue of how to handle the situation in iraq.i have been thinking about this issue for some time now.
- today is a great day for a great day.</td></tr>
<tr><td>Life-
satisfa
ction</td><td>Age Low (-3)</td><td>- i am now officially a junior.
- i'm a college girl. i like college.
- i'm just so excited to go home
- i'm going to the beach with my dad :)
- i'll be leaving soon, so good bye.
- this girl is such a flirt
- i got the best boyfriend in the whole world
- i'll be a senior next year, and this is my second time writing a senior year letter.it's a pretty simple letter, but it's a lot of fun writing it.i'll be leaving for college in less than 5 weeks, so i'll probably write something like that.
- well it's been a long time since i wrote in this.i've just been busy with stuff.i got a letter today from my dad.it was really cute.
- i'm leaving tomorrow at 11pm.</td><td>- my hair is so stupid. i hate it.
- i'm bored and i'm bored and i'm bored and i'm bored and i'm bored and i'm bored and i'm bored and i'm bored and i'm bored and i'm bored and i'm bored and i'm bored and i'm bored
- i hate my sister so much.
- people be so fake on twitter...
- this is why i'm so tired.
- this is my first attempt at blogging so hopefully i don't fuck it up.
- i got my own blog, i'm going to be updating it a lot more than my other blog.this blog will be my personal blog, and i'm going to put my feelings and my thoughts into it.
- i'm so bored... my sister's not on the computer so i'm stuck here.
- i'm so sick of this.i'm so sick of this.i'm so sick of this.i'm so sick of this.i'm so sick of this.i'm so sick of this.i'm so sick of this.
- this is my first blog and i really hope that i don't screw it up.i'm just bored right now, but i'm going to try to write something interesting.</td></tr>
</table>

	**Age High (+3)**	- my daughter just turned 6 today. she is so beautiful, and she is so sweet.<br>- it's all good, it's all good, it's all good! peace, love, and happiness to all.<br>- i am so thankful for my family and friends<br>- i'm thinking of buying a pair of jeans.<br>- happy birthday to my big brother.<br>- i just heard the most beautiful news.<br>- ok, so the new york times has this to say about my new book: "the new york times review of books says of "the art of the possible" "a book of great wit, insight and wisdom."<br>- i am going to have so much fun at this wedding.<br>- i need to get out of the house more often<br>- i need to get my life in order	- i hate when people get on my nerves<br>- i'm tired of hearing all the bullshit.<br>- i'm getting old and fat and i'm losing my mind<br>- i'm sick.i've been feeling really sick all day.i think i'm coming down with a cold.i'm really tired too.i haven't been to bed all that late.<br>- my back is killing me. i hate this job.<br>- this is the kind of shit i hate<br>- i'm tired of the bullshit. i'm too nice for shit.<br>- i'm sick as a dog.i can't keep the kids out of the hospital.i'm not getting paid.i'm not feeling good.my head hurts.my throat hurts.i'm cold.<br>- i'm so sick of hearing this.<br>- i have a big ass headache

Randomly selected text generation for Big Five Personality without prompts, trained on Blogs dataset only

	High (+3)	Low (-3)
**Ope**	- i've been working on my website for a while now.i've been trying to get it to look nice and professional, but i've been having a hard time.i'm not quite sure what i'm doing.<br>- i am so inspired by the work of the people in my life.i have learned so much.and i have been so moved.i am not sure what i will do with this knowledge.<br>- i've been meaning to write this for a while, but i've been busy with work and the rest of my life.i've always been fascinated by the idea of time, and it's meaning.<br>- i've been thinking about the idea of 'the blog' and the idea of 'the blogosphere' and the idea of 'the blogosphere' as a whole.i've been thinking about the idea of 'blog' as a verb, as a noun, as a way of life, as a way<br>- i've been doing a little research and found that the word "blog" is actually derived from the french word "blogueur."a blogueur is a person who writes a blog.in french it is a blogueur.<br>- well, i've been busy, but not too busy.this week i have: -finished my book, which is a collection of poems and stories, and is called in the midst of the dark, and it's about a bunch of crazy things that have happened to me in my life, and how<br>- i've been working on a project with my friend and colleague, dr. scott smith.this project is a study of the relationship between the mind and the body.it's a book project, and we're trying to find a publisher for it.<br>- i've been reading the following book by the author of the secret.this book is a little different from the other books on the subject.the author is not a scientist or a psychologist.<br>- so, i've been thinking about how i can get this blog to be more interesting to people.it's a little difficult to write about things that i'm not interested in.<br>- i'm in the middle of writing a paper on the subject of "the role of the artist in society" and i've come to a conclusion.i've always thought that art was a necessity for the human race, it's what makes us human.	- i went to the park with my brother, my sister and my grandma and uncle.it was fun.i had a good time.but when i got home i couldn't sleep.<br>- i have no time for this blog.i am going to bed soon.but i wanted to say that i am glad that the olympics are over.i hope the usa wins gold in the basketball.<br>- i have a bad back.<br>- today was the first day i didn't have school.yay!i was so happy.but today was also the first day i was in school and it sucked.it was a bad day.<br>- i am going to see my best friend tomorrow.she's coming to stay with me for a few days.it is going to be good.i am excited.i can't wait.<br>- well i had to go to school today.i was bored and i didnt get to go to the movies with my friends.but i got to go to the mall and get some clothes.<br>- so i have been home for like 2 days.well, i was home 2 days ago.yesterday, i went to a party.i had a good time but i got home late and i was like "oh i have to go to school in the morning".<br>- i don't think my car will make it to the game.it needs a new alternator.it's not worth it to take it to the shop because i can't afford it.<br>- well today was pretty good.i had a good day.went to a friends house to watch a movie.the movie was "the day after tomorrow".it was a good movie.<br>- today i went to school with the hope of getting my hair done.i have been wanting to get it done for a while now but my mom said i would have to wait until my birthday.
**Con**	- i have a lot of work to do today.i have to get all my projects done and finish my report and get them ready for presentation tomorrow.i have a lot of work to do today.<br>- this is the first time i have been in the office for 2 days in a row.my first day of being on the job was a complete disaster.<br>- i have been so busy lately, i have been working 50 hours a week.i have been working 5 days a week.i am starting to feel the effects of it.<br>- well, it's been a hectic couple of days.i was supposed to be in chicago all week but i ended up staying an extra day.i was supposed to be working 40 hours but i ended up working 64.<br>- i have to admit, i am a little behind in my work.i am a little stressed.i am a little tired.but, all in all, i am a little excited.<br>- i was at work this morning and a young woman came in to see me.she told me she was in the process of buying a house.i offered her a hug and some encouragement.<br>- i am so very stressed out!i have been working like a dog for the past few weeks and i still have a mountain of work to do.it is not a huge mountain, just a mountain.<br>- it's been two long weeks since i have been able to post to my blog.i have been very busy with work, and the holidays.i am glad to be back.<br>- today was a long day.i had to work a 12 hour shift and then i had to get up and go to the gym for a half hour.it is a long day of work.<br>- the end of the day i just finished my day.i am now on my way to the gym and then i will be going to a doctor's appointment.	- i'm bored and i'm listening to some good music.the best thing about this is that i'm listening to it while i'm sitting at the computer and i can watch tv and do whatever i want.<br>- i'm bored and i'm not gonna go to sleep anytime soon.so, i'll just post a song.i'll be listening to it all night.and i'll be bored.so here it is.<br>- i just realized that i have never posted a picture of myself. that's pretty sad. i'm going to get on the computer now and find some pictures.<br>- i'm not even gonna try and explain what's up.i'm gonna just post a picture of the most beautiful girl on earth.you'll figure it out.<br>- ok, so i've got a new phone and it's a pretty cool phone.i can send pictures and stuff.i'm pretty bored so i'll post pics later.<br>- so yeah, i'm bored, so i thought i'd post something, just for fun.i'm not gonna be on all day, just thought i'd post something.so yeah, i'm not gonna be on, but i'm not gonna be on for a while, so yeah<br>- i'm bored... so i thought i'd post some pictures of myself...<br>- i think i'm going to get a new computer. i'm tired of my crappy old one.<br>- i just got off the phone with my mom, and she's kinda pissed.i'm not sure why, she's just mad at me.i don't know why.but i'm not sure what to say.<br>- i'm bored and i've got nothing to do.i don't really know what i'm gonna do.i'm gonna go to sleep.i'll be back later.i'll probably have something to say.
**Ext**	- i had a good weekend.i'm a little tired though.the party at my friend's house was so much fun.i was there from 7-11:30.we were in the basement.<br>- my dear, my dear...my dear... my dear, my dear...my dear... my dear, my dear...my dear...	- i have finally started reading the hunger games.i started reading it on monday and haven't stopped since.i finished the book in 3 days.i'm not sure if i'm going to finish the second book, or just start reading book two in a few days and finish it at a slower

	- so last night we went to the beach.it was a great night.we met some really nice people.we went to the movies and then had dinner at a restaurant called "the beach house" in newport.<br>- i'm so excited.my friends are coming to town.i'm going to have a party for the first time in 3 years.it's going to be fun.i'm gonna drink.<br>- i am so excited.i can't sleep tonight.i have to go to the beach.i'm going to be in the sun all day and i'm going to be in the water all day.<br>- i'm gonna be a big girl and have a party for my birthday.<br>- well, i'm off to work. i'm going to go see my cousin for her birthday. it's going to be fun!<br>- ok, i'm gonna be in a big trouble.i'm going to miss my 3 days off to go to my best friend's wedding in the philippines.i'm gonna miss her wedding and the 2nd day of the wedding reception.<br>- hey everyone.i'm at my friend kristi's house right now.we are having a slumber party.she invited a friend, and we are all hanging out.i'm so tired.<br>- i'm going to the mall tonight.i'm going to buy a shirt.i'm going to be the only girl wearing a shirt.i'm going to be the only girl wearing a shirt and looking like a girl.	- i found out that the reason i had been unable to access the internet for a couple of days was because i didn't have a broadband connection.i didn't know this until the technician told me.<br>- so i didn't get to post yesterday for the usual reason.i was in a car accident.it wasn't my fault, but i was in the wrong place at the wrong time.<br>- i didn't realize how much i needed this until i actually had it.i'm finally back in school full time, after 4 years of part-time.i'm not going to lie, i've had a lot of trouble trying to adjust to the new schedule.<br>- i am so happy that the weather finally cleared up, the air has finally become crisp and cool, and the sun is back.i am so happy.but, i can't help but think of the snow.<br>- today was a pretty interesting day.i had a lot to do, and it took me a while to get done with everything i had to do, but i finally got it done.<br>- i have been playing around with the html code for a while now.i finally got it to do what i wanted.i just wanted to get it done so i can get back to writing my paper.<br>- the weather here is really nice today.it's warm, sunny, and breezy.the only thing that is bothering me is that i have a paper due today.i'm not sure what i'll be doing to study for it, but i know it's going to be a challenge.<br>- well, i finally managed to get the site up and running.i had a lot of problems with the server, which finally came to a head yesterday.i didn't know what was wrong with the site, so i had no idea what to fix.<br>- i have been trying to find out why my blog is not working.i have tried to find the problem for a couple of days now.i have found that the blog is not working because the template is not working.
**Agr**	- i just got a new blog.i love it so much!i have a feeling it'll be a lot better than this one.i'll have to see how it goes.<br>- i haven't been writing in a while.i've been so busy.i've been busy with school, i've been busy with friends, i've been busy with life.i've been busy with a lot of things.<br>- so, i've been busy lately.i've been busy, and i've been busy.i've been busy making my life better and i've been busy making my life worse.i've been busy being me, and i've been busy being not me.<br>- hey guys!i hope that you all had a great weekend!mine was really fun and relaxing.i went to a wedding on saturday and i was the only one who was there for my friend.<br>- i'm back!i'm back!i'm back!i'm back!i'm back!i'm back!i'm back!i'm back!i'm back!i'm back!i'm back!i'm back!i'm back<br>- i've been busy.i've been busy.i've been busy.i've been busy.i've been busy.i've been busy.i've been busy.i've been busy.i've been busy.i've been busy.i've been<br>- i am so tired. i had a really fun day, but i am tired. i have a really good friend and i've never had one so i am really happy.<br>- today was pretty good.i got up, took my meds, and got ready for my day.i got a lot done.i got my room cleaned, did some laundry, got my bed made, and made some lunch.<br>- i'm so happy today!it's been a long time since i was that happy.it's like a whole new life.today i got a lot of letters from friends and family and i felt so loved.<br>- i just got back from the most amazing day.i spent it with my best friend.she's so special to me.we had so much fun.we went and got some food and sat on the grass in the park and ate.	- so i don't know how to spell it but i'm going to say it anyway. that's right, i'm talking about the fucking fucking fucking fucking fucking fucking fucking fucking fucking fucking fucking fucking fucking fucking fucking fucking<br>- it's not a conspiracy theory, it's a fact.i am an american.this is my country.i have the right to vote, to go to war, to own a gun, to drink beer, to smoke, to have children, to have sex, to wear whatever the fuck i want,<br>- i don't know what it is with me and the women.i just can't seem to catch a break.i'm always getting screwed by women.they're always cheating on me, or lying to me.<br>- i'm in a mood right now.i don't know what it is.i don't even know why.i'm not in a bad mood.i'm not in a good mood.i'm just in a mood.<br>- "i'm not a racist."i hate to say this but i do not believe him.he is a racist, and i'm not talking about the fact that he is a republican.<br>- this was the most ridiculous story i have ever heard.i was watching the news and i heard a story about this woman who was trying to kill her husband.<br>- i don't care what you think, but i will not go to war in iraq to protect the people of iraq from iraq's enemies.the iraqi people are not our enemies.<br>- i'm not a fan of the war, but the iraqis are terrorists.they have no problem killing american civilians.they're the ones who killed that innocent little girl.they're the ones who killed that innocent little boy.<br>- i don't think i can be a lawyer.i don't think i can be a teacher.i don't think i can be an engineer.i don't think i can be a doctor.<br>- i am not a racist, but i am not a racist.
**Neu**	- i'm tired.i'm tired of feeling like i'm not good enough.i'm tired of feeling like i'm not worth the effort.i'm tired of feeling like i'm not good enough to be here.<br>- i am so tired of this whole thing.i am so tired of it.i am so sick of being sick.i am so sick of being sick.i am so sick of being sick.<br>- i am so tired.i am so tired of everything.i am so tired of being sick.i am so tired of being in	- so i'm playing the game "what would you be if you were a fruit?"and the question is "if you were a fruit, what would you be?"i answered "a pineapple."<br>- well, i just got back from the most interesting thing i have ever seen at the beach.this is the first time i've seen a sea turtle.i thought it was a dolphin or something.<br>- "you know, when i was a kid, we used to have this game called 'the big game,' and i

	pain.i am so tired of being alone.<br>- i woke up at 4am today.i had been up since 11pm, but i'd been sleeping on and off since 9pm.i'd had an appointment for 10:30, but i'd gotten to my house at 8:30, and i<br>- i am tired. i am sick. i am in a funk. i am tired of feeling like this. i am tired of having to be the one who makes things better. i am tired of having to make things work. i am tired of feeling like a victim. i am tired of not being able to sleep<br>- i am exhausted and i am sick of being sick of being sick of being sick of being sick of being sick of being sick of being sick of being sick of being sick of being sick of being sick of being sick of being sick of being sick of being sick of being sick of being sick of being sick of being sick<br>- i am so tired.i am so very tired.i am sick of waking up in the middle of the night to go pee.i am sick of not being able to sleep through the night.<br>- i'm tired, and i'm in a bad mood.i'm tired of being tired, and i'm in a bad mood about that.i'm tired of being tired, and i'm in a bad mood about that.<br>- i'm tired.i'm sick.i'm tired of this.i'm tired of feeling like i'm not good enough.i'm tired of feeling like i can't do enough.i'm tired of feeling like i'm just not good enough.<br>- i'm tired.and my head is hurting.and i'm still sick.and i'm still sick.and i'm still sick.and i'm still sick.and i'm still sick.and i'm still sick.	always played as the big man and you always played as the little man and you always had to try to get your big man to the big man.<br>- i'm playing with a simple java applet that i've written.it draws an image on the screen and then draws a circle on that image.it's the simple stuff.the applet is a simple test of a basic java application.<br>- i like the idea of a new language, but i don't know if it's really practical for us to do.<br>- the game that has it all.<br>- i am the man that knows the world, the man that knows the world.<br>- i'm a man of the sea i'm a man of the sea i'm a man of the sea i'm a man of the sea i'm a man of the sea i'm a man of the sea i'm a man of the sea i<br>- hey, this is my first time to write in this blog.this is a good way to know the other people out there.i am a 26 year old guy and i am working as a software developer.<br>- i am a big fan of the movie "the day after tomorrow."i love the concept of the movie, and i love the cast of the movie.i think it's a great flick!

Randomly selected text generation for Big Five Personality without prompts, trained on Tweets dataset only

	High (+3)	Low (-3)
**Ope**	- i've been working on my writing for over a year now. i'm proud of myself for that.<br>- i'm about to go to the gym and read my book.<br>- i'm so happy to have a full head of hair.<br>- i feel like a different person today...<br>- i'm so happy i'm in my own place.<br>- i'm in the zone for this paper<br>- i've learned to be content with my life and i'm grateful for everything that's happened.<br>- i've been reading the same book for the past 2 weeks<br>- i've never been this stressed about a job interview in my life<br>- i'm in the middle of the 12th floor of the library. i'm not going anywhere.	- i hope you win, i hope you lose.<br>- i want to leave school already<br>- i dont know how to play football :(<br>- i dont want to get up to school tomorrow<br>- i miss my mom. i hope she has a good day today<br>- i hope this day goes by fast<br>- i need a car i dont have one<br>- i need to stop buying so much clothes<br>- i just wanna go home<br>- don't forget to go see my dad tomorrow.
**Con**	- i shoulda ran a few miles today.<br>- my schedule is crazy right now i can't even focus.<br>- i really need to start working out. i'm 50 pounds overweight.<br>- my boss is such a bitch.<br>- my boss is so demanding, and she expects the same level of professionalism from the employees.<br>- i need to be more patient<br>- can't wait for the week to start!<br>- it's the end of the work week. time to relax and prepare for the weekend.<br>- my schedule is so hectic right now i can't even begin to explain<br>- the end of the day is the best part of the day	- i'm bored and i need something to do.<br>- i wanna go back to sleep but i cant<br>- i guess ill just go to sleep<br>- i'll be the best version of myself<br>- i'm just not in the mood for nothing<br>- i'll never be as pretty as you.<br>- i'll be in my room watching the game and i'll be in my pants<br>- i think im going to be a bad influence on my nephew lol<br>- i just wanna be a good person<br>- i wanna be a cool girl but i'm pretty sure i'm a bad girl.
**Ext**	- i wanna see the big band tonight<br>- my life is so fukin crazy right now.<br>- i think im getting the hang of this dating thing<br>- i'm ready for the new year to come<br>- i miss the nights when i could party with no one<br>- i wanna take a trip to a big city<br>- i think i'm gonna go to sleep<br>- i love when girls have big boobs and big ass<br>- i'm gonna be the drunkest person at the party tonight.<br>- i'm ready for this night to start	- i've been reading a lot of books lately and i think it's time for some new reading material<br>- i can't wait to finish this book<br>- i can't wait to watch the new season of the office<br>- i didn't realize how much i needed that 20 minutes of peace and quiet.<br>- i didn't realize how much i needed my family until i moved away.<br>- i didn't even know it was snowing outside<br>- i haven't been to school in two weeks.<br>- the only thing i want this year is a new computer<br>- i've been reading this book for 3 weeks.<br>- i didn't have time to finish my paper today. i'll finish it after i finish my homework.
**Agr**	- i just want a hug<br>- i love the feeling of a great night of sleep<br>- i love the way your heart works<br>- i've been so happy today :)<br>- my mom's voice is just so beautiful<br>- i've never been so excited for a concert.<br>- i've been feeling pretty good today.<br>- my life is a song, and i'll sing it<br>- i made a good friend today.<br>- i just want to hug you, hold you tight.	- damn i wish i had a gun to shoot these n****s<br>- i wish my ass was on vacation!<br>- i swear i hate the word ass<br>- damn my n**** got a 93% on the test<br>- wtf is wrong with people today<br>- this n**** is so damn annoying<br>- damn this n**** was a fucking idiot!<br>- wtf is this bullshit with these 40k's?<br>- i don't fuck with no n****s with no hoes.<br>- i don't even like talking to people, that's why i be on the internet lol.
**Neu**	- i'm tired of being tired.<br>- i can't stop thinking about my mom<br>- i'm tired of being sick. i'm tired of being in bed. i'm tired of feeling like crap. i'm tired of being	- imma eat this food, then go play ball<br>- it's a good time to play some games.<br>- it ain't no fun in the jungle

	sick.<br>- i can't sleep at night. i just keep thinking about how much i hate my life<br>- i feel like i should be in bed right now<br>- i can't wait to sleep in the bed that's in my room<br>- i can't even sleep. i'm so stressed out.<br>- i'm tired, i need to go to sleep.<br>- i'm tired of being tired of being tired of being tired<br>- i'm tired of being hurt	- i need some new glasses.<br>- bout to eat some chicken and fish<br>- the real question is, what's your favorite type of food?<br>- lol this man is funny as hell lmao<br>- i ain't gotta play the role of no man<br>- this n**** just ain't got a job smh<br>- i ain't got time for no fake ass n****s

S2.A. Human experts evaluation for Big Five Personalities (without prompt)

**Instruction:**	There are in total 10 sets for each variable. For every set, 5 messages are generated by Psychgenerator at 3 positions: High, Neutral and Low. However, human evaluator (you) don't know which position the texts were generated from. Human evaluators need to read all the texts of A, B and C and match them to the correct position they were generated from.	Below are the 10 sets for evaluating for **Extraversion** variable.

Set 1		Your answer		Correct answer	Human expert 1	Human expert 2
**A**	i finally got the computer fixed.i think it was a loose connection on the power supply.it was a good thing toc<br>i was looking at my calendar for the month and realized that i haven't done much of anything for the last tw<br>i don't know what i did wrong this time, but the game just won't load for me.<br>so, i just finished reading the first book of the series, "the hunger games".it was a good read, but i was a littl<br>"the only difference between a computer and a dog is that the computer doesn't have to be told what to do	**Low**	**?**	**A**	**A**	**C**
**B**	i love my best friends.i love my best friends' boyfriends.i love my best friends' boyfriends' parents.i love my<br>i'm so excited about tonight, it's going to be a big night.i'm going to see the new "love actually."it's going to<br>i'm just gonna say it, i'm a total slut.<br>hey everybody, i just wanted to let you know that i will be having a big party on friday, august 19th from 7:0<br>so i had a good weekend.i saw my friends, i went to the beach, i saw a movie.i was in a good mood.i was dri	**Neutral**	**?**	**C**	**C**	**A**
**C**	i'm so glad that i am no longer in a relationship. i'm a better person when i'm single. i can do anything i want.<br>well, i've finally decided to go to church.i have been going to church for a few years but i have never really g<br>i was going to post about my trip to the mall today, but i think i'll just post about the whole thing instead.i we<br>well i am back home, and it is a good thing.i had a good time at camp.i am glad that i went, but i am glad to b<br>so i am sitting here and i am wondering why the hell i have been so lazy.i mean i have not done any work for	**High**	**?**	**B**	**B**	**B**

Set 2		Your answer				
**A**	well, i finally did it.i got my computer hooked up to the internet.i had to go through a lot of trouble to get it<br>it's not a good sign when you start reading the manual and you don't understand a single thing.<br>i found a way to do it.<br>i've been working on some new stuff.it's been a while since i've had a chance to do any serious programmin<br>i was able to finish the first chapter of the hunger games	**Low**	**?**	**A**	**A**	**A**
**B**	i don't think there's a person in the world that would have predicted that i would be a single mom.i have alv<br>i'm so happy that i don't work on saturday.it's a nice change.today was a nice day.i spent it with my girlfrien<br>the first thing i want to say is that i love this blog.i've been reading it for a while and i think it's the best thin<br>i'm going to have to be the only person to go to work on sunday and i'm not looking forward to it.<br>the world's a better place for people to be nice to each other	**Neutral**	**?**	**B**	**B**	**B**
**C**	so i am in love, or at least i think i am.i am in love with my best friends boyfriend, and i am really happy abo<br>well, i am back from the best weekend ever.i had a blast.it was so funny.i had a blast.i was so drunk.i had a b<br>"you know, you should call me tomorrow."<br>i'm just a girl from the country that loves a boy from the city.<br>i'm so excited i can't even stand it!	**High**	**?**	**C**	**C**	**C**

Set 3		Your answer				
**A**	the night is young and the party is just beginning<br>ok, so i'm not really that interested in what people wear.i'm just really interested in their personality.i know, i<br>the boys and i are going to the movies this weekend.it will be our first time seeing a movie together.we've be<br>"i want to be with you and only you"	**Low**	**?**	**B**	**B**	**B**

	ok, so i'm not a very good blogger, but i'm still blogging.i'm going to a party tonight, and i'm gonna get hamr					
B	i've been playing around with the idea of getting a new pc. it's about time.<br>i've just finished my first ever book, the "the secret" by rick santorum.it's a book on the law of attr<br>i have a lot of things to do today.<br>i found the time to watch the first episode of the last season of the office<br>i found a book i read a long time ago, but i never finished, and i thought it might be time to finish it	Neutral	?	C	C	C
C	i'm not a fan of the word "no" but i have to say i love it right now. it's been a while since i've gotten to use it.<br>well, i'm here in the city of angels and i'm not too happy about it.i've been here about 30 minutes and i've alre<br>this is the first time in my life i'm so excited about going to work.i'm not even sure what it is i'm looking forv<br>i just want to be happy, that's all.<br>i'm so happy that my brother is going to be home today.i'm not sure how much he'll be able to help with my v	High	?	A	A	A

Set 4		Your answer				
A	i think my mom was right. i need a new phone.<br>i've been trying to get ahold of the people at the university, but they're all on break, so i haven't heard back<br>i'm reading a book on how to build your own website.i'm trying to find out what it is about the internet that<br>"a good book is the greatest of all the luxuries.<br>"a lot of people don't understand the value of the computer and what it's capable of, and i think that's a mis	Low	?	A	A	A
B	i am going to be a big girl and get my hair done today.<br>it was so cold this morning.i had to wear my jacket even though it was 8:00 am.i had to take a shower becau<br>i have to say that this is the best thing i've ever written.i just love it.it is so true.i have been in the same posi<br>i have to admit i'm really starting to miss the old me...i miss the old me who didn't have to worry about the<br>i'm not sure if this is the right place to put it but i was wondering if anyone knew of a good place that would	Neutral	?	B	B	B
C	i'm ready for some good sex tonight.<br>i love how i get drunk with my friends<br>i love the way you make me feel<br>i love my friends.i love my friends so much.i love my friends so much.i love my friends so much.i love my f<br>my baby daddy is so in love with me...	High	?	C	C	C

Set 5		Your answer				
A	i love my girls. they make me feel so good.<br>i'm so excited! tonight's my 20th birthday! woohoo!<br>well, i'm a little nervous.i'm going to be seeing the band at the 4th of july party.i have no idea what t<br>well, tonight was a good night.i got to hang out with my best friend, and we went out to dinner and went to th<br>i just wanna get drunk with my girls	Low	?	B	B	B
B	well, the computer is finally fixed.it is a bit of a pain to get the hard disk in, but once it is in, it is easy.i don't<br>i've been working on the new version of my site for a while.the old version is still up, but it will be going awa<br>the weather is getting warmer and that means spring is on the way.this means i can start working on my nev<br>it's not just the fact that i haven't been able to get online, it's also the fact that i can't get online at all.<br>so the computer has been working.finally.it was a slow process getting it up and running.i had to do a bit of	Neutral	?	C	C	C
C	"my ""friends"" are the ones who make me happy. they make life worth living. ""friends"" are the ones who<br>i'm so proud of myself for making it through another day.i have to say it's been a rough week, but i guess that<br>i'm so glad i'm not the only one who's been in love with this show since it first aired.i'm not a huge fan of the<br>i'm not sure why, but i've had a really hard time sleeping lately.i'm not really sure why, i mean, i'm still in my<br>i have no idea what i'm doing with my life.	High	?	A	A	A

Set 6		Your answer					
A	well, i'm going to go see the show tonight and i'm not sure what i'm going to do with myself.i'm going to see<br>i'm so excited for the new season of the bachelorette!<br>so im just gonna go to bed...<br>i'm so drunk and i have a big day tomorrow.<br>i'm not going to be at the party on wednesday night.i'll be in my room at the hotel.it's going to be a party, b	Low	?		C	C	C
B	"when you're in a good mood, you want to talk to someone.when you're not in a good mood, you don't war<br>i have a friend who is a great cook.i have eaten at her place, and i am always in awe of the way she cooks.i a<br>i just realized that i have not blogged for over a week.i'm sorry.i was busy.and tired.but now that i am not, i<br>i got my first tattoo.it's on my back and it's a phoenix.i got it from a guy in the tattoo shop i went to.it was a<br>i think i'm ready to start a blog.i'm a 25 year old single mother of two.i've been in a bad place for a while an	Neutral	?		B	B	B
C	i'm not sure why i'm reading this, but the book i've been trying to read for a while, and haven't had time for, i<br>i'm back and i have some good news for all of you.the new computer should be in a week or two.we'll see ho<br>so i've finally gotten around to getting the computer to do what i want it to do, and that is to post.i've got to g<br>i was able to find the time to read a book in the last 30 days.<br>i'm going to be in the dark for a while now.	High	?		A	A	A

Set 7		Your answer					
A	it's not even 7am and i'm already bored<br>i just wanna be the one to make you smile<br>i'm going to have to make a new blog.i'm getting bored with this one.it's too much work to keep up with.so,<br>i have a feeling that this will be a great year for me.i've been thinking a lot about the past and how i was not<br>i just wanted to say a few words about my brother.he is a very good man.he is very good at what he does ar	Low	?		C	C	C
B	i'm a little nervous, but i think i'm going to have a good time.i'm not sure if i'm going to make it to the party<br>i'm gonna get so pissed if i wake up and he's gone.<br>so i'm in a car with my cousin and his gf, and they're talking about how much they love each other, and how<br>the night is young.i had a great time last night.i hung out with my old friends from highschool.i had a great t<br>the night is young, and i'm drunk.i'm not sure what i'm drinking but it's a lot.i'm not sure what i'm doing but	Neutral	?		A	A	A
C	i have a lot of thoughts on this.i have been reading about some of the recent discoveries in physics<br>so i've been reading about the new windows 2000 server.the new version of windows server has a lot of new<br>i just finished reading "the last unicorn" by peter david. it's a great book. i'm not sure how long it will take m<br>"i've been thinking about a good book to read, but i have no time to read.i've been busy, i've been playing cor<br>well, i finally finished my first book.i finished the book on wednesday, but it took me until yesterday to finisl	High	?		B	B	B

Set 8		Your answer					
A	i think i just found a new favorite band.i've been listening to them for a few months now and i'm just now ge<br>i'm just gonna say it: i'm not a big fan of christmas music.i like the songs that have a good message, but i do<br>i'm a big fan of the book "the art of war" by sun tzu.it's an ancient chinese text that has been used by many<br>so i'm on the bus and i see this kid sitting in the back.he looks like he's in 8th grade.he has a very serious fac<br>so today, i'm sitting on the bus, reading the newspaper.and i'm thinking about how much i love my life.i hav	Low	?		B	A	A
B	"it's not that i am unable to think of the right words, it's just that i can't think of the right words.i know this<br>i don't know why i didn't get a chance to write a post about my trip to the library.i guess i was just too busy<br>i have to finish the homework i started yesterday.i've been reading the first few chapters of "the last battle"<br>this is a good place to post my thoughts on a couple things.the first thing is a question.what is the purpose c	Neutral	?		A	B	B

	the only thing i have to complain about is the lack of a 2nd screen.					
C	i need to get my nails done, i'm so bored, and i'm getting a new tattoo tomorrow!<br>i just wanna be a girl again.i miss you so much.i wanna be a girl again.i want to go back to high school.i wan<br>i need to go out with my friends tonight.<br>so i just got off the phone with my sister and i am so excited i could scream.i just got a job at the new york ci<br>i wanna go out for drinks tonight	High	?	C	C	C

Set 9		Your answer				
A	the only reason i'm still playing the sims is to get the new expansion pack<br>well, i finally got a chance to get my hands on a copy of the new version of microsoft word for mac.i was a li<br>i finally found my way out of the forest.i was lost for a while, but then i found the right path.now i can see th<br>so, i've been playing around with my new computer for a few hours now.i finally got all of my old stuff trans<br>i'm not going to be able to find a job until i have my own laptop. i need a computer to do all my research for	Low	?	A	A	A
B	"you know you're in love when you wanna go out with your best friend's ex-boyfriend"<br>i love how the girls are always the ones that wanna talk about it<br>i wanna go to the beach with a girl<br>well i went to the mall today to meet up with my boyfriend and i got a whole bunch of stuff.im sooo happy.i<br>i love you guys and i love you all so much. i miss you all so much.	Neutral	?	C	C	B
C	i just saw a guy with a big ass tattoo of a dragon on his arm, and i'm like...i'm not gonna judge you for that, b<br>i'm so tired of the way i feel.i'm tired of the way i act.i'm tired of the way i live my life.i'm tired of the way i<br>i'm so glad i'm not a high school kid anymore.<br>i'm not the biggest fan of the new twitter layout, but i'm getting used to it.it's nice to have the option to have a<br>i'm just glad i don't have to deal with the 1000s of questions i get on here about what to do with my life. i'm g	High	?	B	B	C

Set 10		Your answer				
A	i've been a bit busy this week, and i've been thinking about the whole issue of what i am going to do next ye<br>i'm not sure how i feel about this.i was thinking about what i should do with my life, and i realized i'm not su<br>"i don't know why i'm so obsessed with ""the one ring to rule them all."" but i am. i love it."<br>"i have to get out of this room!i'm going to die if i don't get out of here.it's so stuffy in here!i can't breathe!i<br>the only thing that can keep me from getting a tattoo is that i have a job.i don't want to give up my day job a	Low	?	C	C	B
B	i'm so excited for tomorrow night, i'm going to be a bridesmaid in my friends wedding, it's going to be the g<br>the way that i'm goin to sleep tonight is the way i'm goin to wake up in the morning.<br>"i'm so in love with a girl i can't even say her name." -- "i'm so in love with a girl i can't even say her name."<br>i wanna be drunk with my bestfriends and have the best time<br>the guys are all here for the party and i am all alone with my 2 dogs.i am going to have to have a drink.and i	Neutral	?	A	A	A
C	if you're a blogger, you might want to read this.i'm not sure if it's a good thing or a bad thing, but it's worth a<br>the weather in my city is so nice today.<br>i think i got my hands on a few copies of the new the matrix movie.i haven't seen the first one yet, but i'll see<br>i've finally found my way around the internet.i've been using this computer for about a month now, but i neve<br>i'm reading the book "the art of the good life" by john kerry, and one of the points that really hit me is how m	High	?	B	B	C

S2.B. Human experts evaluation for Big Five Personalities (with prompt "I like to")

**Instruction:**	There are in total 10 sets for each variable. For every set, 5 messages are generated by Psychgenerator at 3 positions: High, Neutral and Low. However, human evaluator (you) don't know which position the texts were generated from. Human evaluators need to read all the texts of A, B and C and match them to the correct position they were generated from.	ow are the 10 sets for evaluating for **Extraversion** variab

Set 1		Your answer		Correct answer	Human expert 1	Human expert 2
**A**	I like to think that i'm a very patient person, but sometimes i get a little impatient.i don't know why, but i do.i was driving do	**Low**	?	C	C	C
	I like to go out on a limb and say that i am an optimist.i believe that things will work out and that i can make things work out					
	I like to be able to do everything myself.i don't like having to depend on other people.i don't like having to ask for help, and					
	I like to think i'm a pretty good guy, but i'm not perfect.i have had a few mistakes in my life and i'm not proud of them, but i'					
	I like to think of myself as a liberal, but i don't know if that's the truth.i don't know if i'd call myself a democrat, but i do thin					
**B**	I like to have a good time. i like to get drunk. i like to get laid. i'm not a prude, i'm just not a whore.	**Neutral**	?	A	A	A
	I like to party and drink with my friends.					
	I like to party and dance.i like to have fun.i like to be out and about and i like to drink and i like to be with people.i like to be					
	I like to wear a skirt to bed. i'm just sayin.					
	I like to think of myself as the girl who always gets her shit together.					
**C**	I like to read about the history of the internet.i find it interesting to know what happened before i was born.this week, i read a	**High**	?	B	B	B
	I like to think i know a thing or two about history, and i'd like to think i can see a little bit into the future.but i don't think that					
	I like to think of myself as an intelligent person, but sometimes it's hard to remember that.					
	I like to think of myself as a pretty good writer, at least when it comes to writing for myself, and for my class.but i'm not real					
	I like to read and write, and have a lot of books in my library.i have to find time to read them and i have to find time to write					

Set 2		Your answer				
**A**	I like to think that i can be a little more open about my thoughts and feelings on the internet.i don't like to say what i think o	**Low**	?	B	B	B
	I like to think that i am a nice person.i don't like to be lied to.i like honesty.i like to know where i stand.i like to be treated like					
	I like to think of myself as a liberal.i think i'm a liberal, and i think i'm a pretty liberal one.but i'm also a conservative, and i lik					
	I like to think of myself as a person with a good sense of humor, but when i was a kid, there were some things that just made					
	I like to think that i am a pretty easygoing person.i'm not one of those people who gets all bent out of shape about things th					
**B**	I like to read.i've always liked to read.when i was a kid, my mom read to me every day.she would read to me for hours and h	**Neutral**	?	A	A	A
	I like to think of myself as a good person.					
	I like to read a lot.i read books in my free time, and i've been doing it since i was a kid.i think reading is a skill that is importa					
	I like to think that i'm a pretty decent person.i don't have a lot of friends, but those that i do have are pretty good people.i'n					
	I like to think i've gotten better at this whole blogging thing, but i'm still not sure.i'm not sure if i'm doing this the right way.i					
**C**	I like to drink. i like to party. i like to have fun. i love my family. i love my friends. i love my life.	**High**	?	C	C	C
	I like to think that i am a woman, but sometimes i'm not sure.					
	I like to get my drunk on with the boys.					
	I like to wear my heart on my sleeve. i'm a big softy.					
	I like to wear my heart on my sleeve					

Set 3		Your answer				
**A**	I like to read a lot.so, when i get the chance, i like to read a lot.i'm reading right now.in a way, it's like i'm in schoo	**Low**	?	A	A	A
	I like to think i'm a pretty good writer, but i'm not perfect.					
	I like to watch the world go by.					
	I like to write in the mornings. i find that it's easier to focus on writing, and i don't have to worry about distractior					
	I like to think of the internet as a place where people can connect and exchange information.that's what it's supp					
	I like to think i'm not a racist person.but i have to say, the way i was raised, i don't think i could be.i have been ar					

**B**	I like to think of myself as a good person.i try to be honest, i try to be fair, i try to be helpful.but sometimes, i am<br>I like to think that the world is a pretty good place.i'm not saying that everyone is a saint, but i'm sure that most<br>I like to be able to look at my phone and see all the things i'm not doing.<br>I like to make my own decisions, and that's just the way it is.	**Neutral**	**?**	B	B	B
**C**	I like to have a party<br>I like to party, but i'm not a party animal.<br>I like to go to the mall and get a drink with my girls<br>I like to have sex in the morning.<br>I like to go out and drink and have a good time.	**High**	**?**	C	C	C
**Set 4**		**Your answer**				
**A**	I like to think i'm a little bit of a nerd, but this is my first time ever using a blog, so i don't really know what to expect.i'm just<br>I like to think i'm an intelligent man, but sometimes i just can't help but wonder... why do people do the things they do?why<br>I like to think that i'm a decent person.i don't think i'm perfect, but i'm trying to be a better person.i think that i'm a very go<br>I like to think i'm a pretty good person. i'm not a perfect person, i'm not perfect at anything really, but i try my best to be a g<br>I like to think i'm a good person.i know i'm not the smartest or the most beautiful, but i try hard to be a decent person.i hav	**Low**	**?**	B	B	B
**B**	I like to think i'm not as stupid as the other people here.i've been using the internet for years, and i've never had problems v<br>I like to think i'm a pretty smart person, but i'm still learning.<br>I like to think that the universe has a plan.it's just that it has a lot of different plans and we are not supposed to figure out w<br>I like to watch movies.it's a habit i've picked up from my mother.she used to tell me when i was a child to watch the movie c<br>I like to read books about books.	**Neutral**	**?**	A	A	A
**C**	I like to go out and party, and i like to get drunk, and i like to do it all night, and i like to get a little wild, and i like to get a lit<br>I like to think that i'm a pretty good person.i'm a loyal friend.i am not a mean person.i'm a person who loves and who is loved<br>I like to wear my hair long.i like to wear my hair long because i like to feel like a woman.i like to wear my hair long because<br>I like to drink, i like to party and i love the boys.i love the boys and i love the girls, but i love the boys more.i like to party an<br>I like to be the center of attention.	**High**	**?**	C	C	C
**Set 5**		**Your answer**				
**A**	I like to think i'm a pretty good person, but i've done some pretty shitty things in my life.<br>I like to make the point that i am a liberal but not a socialist.i believe in individual freedom and the rights of people to do wh<br>I like to think of myself as a pretty good guy.i'm not perfect, but i don't go around hurting people.i have had people tell me t<br>I like to think that i'm a good person.i'm not perfect, but i try my best to do the right thing.i try to be the person i want to be<br>I like to go to the beach.i like to lay on the beach and stare at the ocean.i like to lay on the beach and watch the sun come u	**Low**	**?**	B	B	B
**B**	I like to think that the only reason i can read so much is because i don't have to read for school.<br>I like to read about things that are happening in the world, but there are some things that i don't want to read about.i don't<br>I like to use my computer to play games.<br>I like to watch movies and read books.<br>I like to think of it as an interactive puzzle.	**Neutral**	**?**	A	A	A
**C**	I like to have fun.i like to be spontaneous.i like to be silly.i like to go out and have a good time.i like to laugh.i like to smile.<br>I like to go to bed at 10:30 and wake up at 6.<br>I like to get drunk with my friends.<br>I like to go out and have a good time.<br>I like to get dressed up.i'm not gonna lie.i like getting dressed up and going out.i like dressing up for the night.i like getting d	**High**	**?**	C	C	C
**Set 6**		**Your answer**				
**A**	I like to be able to say that i'm the only person on earth that can understand your pain.<br>I like to think that i am a good person.i am very generous with my time, i am very kind to people, i am very caring to people.<br>I like to be the first to say that i'm not perfect.i'm not a perfect person, and i'm not a perfect parent.i do not think that i am a	**Low**	**?**	C	C	C

		Your answer				
	I like to be the one who is there for people. but sometimes i just want to be left alone.<br>I like to think that i'm a good person.i'm not perfect, but i don't lie and i don't steal.i'm honest and i try to be fair.i don't chea					
**B**	I like to go to a party, but im not gonna go to that party...<br>I like to wear my hair up.<br>I like to go to the party when the party is at my house.<br>I like to party, but i dont wanna drink<br>I like to call it a night	**Neutral**	**?**	A	A	A
**C**	I like to read the news every morning.i do this because i like to be informed of what's going on around me.i don't have a favo<br>I like to read and i like books.i've never had a computer, so i haven't been able to access a lot of the information i would like<br>I like to think i'm not a big fan of any of the seasons, but i have to admit, i'm starting to like this one.<br>I like to think i'm a pretty smart person.i'm not sure why, but i can always seem to find the right answer to any problem i com<br>I like to think that i'm pretty good at reading people.i guess that's why i don't have much trouble getting along with people.i th	**High**	**?**	B	B	B

**Set 7**		**Your answer**				
**A**	I like to party and get all fucked up, but i also love to sleep.<br>I like to wear a lot of make up and have a lot of hair.<br>I like to drink a lot. i'm a little tipsy tonight.<br>I like to call you up and talk to you all night.<br>I like to party, im a party girl.	**Low**	**?**	C	C	C
**B**	I like to think i'm a good person.<br>I like to think that i am a pretty good person, i try to do the right thing most of the time, and i try to help others in need.<br>I like to go to the library and just sit in a corner and read a good book, and then come back and do my homework.i don't like<br>I like to think that i'm a pretty good person. i try hard to do the right thing and help the people around me. but i have this lit<br>I like to think i'm a good person... but i'm not.	**Neutral**	**?**	B	B	B
**C**	I like to read.it's a nice way to spend an afternoon.there's a lot to read these days.i've read a lot of books over the years, but i'v<br>I like to think i'm a pretty good driver, but i'll always be that kid with a bumper sticker that says, "don't follow me."<br>I like to think that i am a good person.i try to be.i'm not perfect, but i try to be better.but sometimes i do things that i wish i ha<br>I like to think the earth is round because it's the best way to explain the lack of gravity.<br>I like to write in a word document but sometimes the formatting goes wonky when i try to copy it to the blogger.com.so, i tho	**High**	**?**	A	A	A

**Set 8**		**Your answer**				
**A**	I like to party.i'm going to the party.i'm going to the party.i'm going to the party.i'm going to the party.i'm going to the party.<br>I like to call myself a feminist.i am a girl, and i like to believe i am a feminist.i like to think that i am a feminist because i love<br>I like to think i'm a good girlfriend.i'm a good friend.i'm an awesome girlfriend's girlfriend.i'm a great lover.i'm a great friend<br>I like to have a good time...<br>I like to party but i dont like to party like this	**Low**	**?**	B	B	B
**B**	I like to write, and i like to read.i don't like to do either all the time.i have been trying to get better at writing.the problem is,<br>I like to read a lot.i read a lot, but i don't read a lot of books.i read a lot of web pages, a lot of newspapers, a lot of magazine<br>I like to keep my books organized and in order. i have a book case in my room with all my books and notebooks.<br>I like to use my time in the car to listen to audiobooks and podcasts.<br>I like to think i've been doing well in the last couple of weeks, but i've only just noticed that i've been doing better than i wa	**Neutral**	**?**	C	C	C
**C**	I like to make a joke about how much i love my job, but i'd be lying if i said i don't have moments of self-doubt. i'm a teacher<br>I like to think i'm not a bitch, but i really am.<br>I like to think that my life is going to be the same as it was when i was 13.i'm not going to get married, or have children, or m<br>I like to think i'm a good person.i like to think i'm a good friend.i like to think i'm a good wife.i like to think i'm a good daugh<br>I like to think that i am a pretty good person.i don't like to hurt people or make them feel bad.i try to be as fair as possible and	**High**	**?**	A	A	A

Set 9		Your answer				
A	I like to wear the same thing every day.i don't really like to change it up too much, because it makes me feel like a slut.i have<br>I like to drink.i like to have fun.i like to party.i like to have a great time.i like to be out all night.i like to be out all night.<br>I like to drink and i like to dance.<br>I like to go to parties.i like to dance.i like to drink.i like to be around people.i like to have fun.i like to laugh.i like to be around<br>I like to call myself a big girl now.i'm not a little girl anymore.i'm not going to be a little girl for long.my life is getting so much	Low	?	B	B	B
B	I like to read a lot, but i don't have the time.<br>I like to read about the past, present and future.<br>I like to think about it that way.<br>I like to think of myself as a pretty smart person.i've managed to get through my entire degree without needing to take any<br>I like to watch movies, but they have to be interesting enough to keep me watching.	Neutral	?	C	C	C
C	I like to think i am a pretty good judge of character.i am not perfect, but i have a pretty good idea of how people are and i thi<br>I like to think that i have a sense of humor, but i'm not sure that i do.i am a bit of a dork at times.i like to laugh, but i'm not su<br>I like to think that i'm a good person.i'm not sure if i actually am, but i'm trying to be a good person.i try to do the right thing,<br>I like to think that i'm a good person, but i'm not.i've been in a relationship for 4 years, and i've never done any of the things t<br>I like to be in control. i'm not sure if that's a good thing or not.	High	?	A	A	A

Set 10		Your answer				
A	I like to think that i'm a pretty good person, but i've been thinking about the fact that i haven't done a post in a while.i'm no<br>I like to think of myself as a pretty good cook but i'm not a pro. i'm still learning. i'm always trying new things.<br>I like to say that i'm the most un-girly girl you'll ever meet, and i'm not even joking.i'm a tomboy through and through.i don'<br>I like to think that i can see through the bullshit, but i'm not sure.i'm not sure that i can tell if someone is lying to me or not.<br>I like to think i'm a good person. i try to make the best of every situation. but sometimes, i'm just a bitch.	Low	?	B	B	B
B	I like to think that i have a good sense of humor, but that's not always the case.<br>I like to write in my blog and then read it later.<br>I like to watch the sun go down.<br>I like to think that the universe was created by an omnipotent being.it's a nice thought.but it's not the only one.i have also b<br>I like to think about what it would be like to be able to live forever.	Neutral	?	A	A	A
C	I like to go to the club and party with my friends.<br>I like to drink, i like to smoke, i like to get drunk, i like to get high, i like to get wild, i like to get ugly, i like to get crazy, i lik<br>I like to drink.i really like to drink.i really like to drink.i really like to drink.i really like to drink.i really like to drink.i really l<br>I like to party...but i also like to be a good girl.<br>I like to think that my friends are my family.i love my friends.they're the best.i'm so lucky.and i love my mom.she's the best.i	High	?	C	C	C

S2.C. Human experts evaluation for mental health (without prompt)

**Instruction:**	There are in total 10 sets for each variable. For every set, 5 messages are generated by Psychgenerator at 3 positions: High, Neutral and Low. However, human evaluator (you) don't know which position the texts were generated from. Human evaluators need to read all the texts of A, B and C and match them to the correct position they were generated from.	Below are the 10 sets for evaluating for **Depression** variable.

Set 1		Your answer		Correct answer	Human expert 1	Human expert 2
**A**	i'm going to the beach this weekend.i'm excited.i'll post pictures when i get back.i'm<br>i'm a little late to this game but i love it<br>i am back in the states and have been enjoying the weather here in the north east.i a<br>i have the best girlfriend in the world.<br>i love the fact that my boyfriend is a man	**Low**	**?**	A	**A**	**A**
**B**	my dad is really getting on my nerves<br>so much stress... i'm not going to write about it... i'm going to go sleep. bye.<br>i'm feeling a little better about myself.i've been feeling like crap for the past couple o<br>i'm sick of hearing about the war.i'm sick of hearing about how we're losing the war.i'<br>i'm a little pissed off right now.i'm tired of the bullshit.i'm tired of people who don't c	**Neutral**	**?**	C	**C**	**C**
**C**	i have to go to the store to get some food and stuff<br>my mom is a big fan of the bachelor.i was a little disappointed that she did not invite me to the<br>i'm not a big fan of the new "american idol" format.i like that they have a theme song, but i do<br>i've never really been into sports.i've been a huge fan of the game of basketball, but i'<br>i'm a little bit nervous today.i've got a job interview at 2pm.i haven't had one in a while, and i'r	**High**	**?**	B	**B**	**B**

Set 2		Your answer		Correct answer		
**A**	the best thing about summer is summer school<br>i'm back!and i'm back for good!i had a great time in texas, and i'm so glad to be back<br>the only thing that's keeping me alive is my car and my gas tank<br>i have to say, it was a great day!<br>i think im gonna take a nap	**Low**	**?**	A	**A**	**A**
**B**	i've been having some weird dreams lately.last night i dreamt i was at a wedding in s<br>it's been a while since i last posted, but i'm not sure what to say.i've been pretty busy<br>i've always been a believer in the saying 'you are what you eat'.well, i'm not so sure a<br>it's not a good day to be a fan of the saints.<br>i'm so excited to see the new season of game of thrones.	**Neutral**	**?**	B	**B**	**B**
**C**	i hate having to wake up early.<br>my dad is such a fucking asshole i hate him so fucking much<br>i'm tired of being lonely, i'm tired of being sad, i'm tired of having no one to talk to, i'm<br>i'm sick of being sick. i'm sick of being sick. i'm sick of being sick.<br>i feel so lonely and sad today.	**High**	**?**	C	**C**	**C**

Set 3		Your answer		Correct answer		
	i'm so fucking tired. my head feels like it's going to explode.					

A	i'm so fucking tired of being treated like shit<br>i hate when people try to be nice to me.<br>i hate when my mom gets angry and screams<br>i'm a little scared that i'll never fall in love again	**Low**	**?**	B	**B**	**B**
B	i'm so glad that i got to meet you guys.i've been in love with you for a long time.i love<br>i love my boyfriend. he's so perfect.<br>i want some ice cream and a chocolate shake from mcdonalds<br>i love the new blogger.com!i'm really enjoying the new design.i love the new color sc<br>i'm so hungry i'm ready to eat a whole ass bag of chips	**Neutral**	**?**	C	**C**	**C**
C	i'm not gonna be in the best of moods today. i just know it<br>i don't think i'm gonna be able to sleep.<br>i'm so excited to have a day off. i need to clean my house. i need to do laundry. i need to get m<br>"i'm just trying to be myself"" - kanye west"<br>i'm so happy i'm off for the weekend. i have so much to do!	**High**	**?**	A	**A**	**A**

**Set 4**		**Your answer**		**Correct answer**		
**A**	the fact that you can wear the same pair of shoes everyday and not smell like them is<br>i love my girl and she love me back<br>this is a great site.i love it.it's got some really cool stuff.i'm thinking about getting a b<br>i love the smell of fresh paint<br>i just wanna play call of duty	**Low**	**?**	A	**A**	**A**
**B**	i'm not a fan of the new jersey devils, but i do like their new jersey.<br>i'm not the best at being happy, but i'm not the best at being sad either.i've been thin<br>i'm so proud of my dad for being so brave and strong. he had to have a total hysterec<br>i have been reading some of my old blog entries, and i have to say that it is interestin<br>this is the first time i've been to the library in a week.i have been so busy with school	**Neutral**	**?**	B	**B**	**B**
**C**	i just want to go to bed<br>i'm not feeling too good.i don't feel like writing, so i'll just post a random poem.i think it<br>i feel so alone in this world<br>well, i just got my first bad review ever!i am so sad.i have no idea what i did wrong.but i'm no<br>i'm not sure what i'm supposed to write here, so i'll just write what comes to mind.i'm tired.i'm	**High**	**?**	C	**C**	**C**

**Set 5**		**Your answer**		**Correct answer**		
**A**	i am so sick of being sick, i want to get well.i am so sick of not being sick, i want to ge<br>so tired of this fucking place, it's fucking horrible.i'm sick of all this bullshit.i hate the<br>well, i'm a little worried.i haven't been feeling well.i've been feeling nauseous and ha<br>i'm a little bit tired and i'm a little bit lonely.<br>i'm feeling a bit of a twinge of guilt.my friend is feeling a bit down about a situation w	**Low**	**?**	B	**B**	**B**
**B**	i need a full body massage and a nap<br>my weekend was great.i went out to dinner with the family friday night, then had a m<br>the fact that i could drive home in the snow is amazing<br>i've always been in love with you<br>i love my life right now	**Neutral**	**?**	C	**C**	**C**

C	this is a new blog... i'm not sure what i'll use it for.i'll probably use it mostly for posting music<br>i'm really trying not to get my hopes up too much for this game, but i can't help it.i really hope<br>i'm going to be a dad in december. i'm so excited<br>i'm going to be on a plane for 8 hours. i'm going to be on a plane for 8 hours. i'm going to be o<br>i have a feeling this will be a long one.i'm in a mood to talk about everything i can remember.l	**High**	**?**	A	**A**	**A**

**Set 6**		**Your answer**		**Correct answer**		
**A**	this fucking sucks.i feel like a fucking loser.i hate feeling like that.i'm just a loser.i hav<br>i don't understand why i'm so tired.i'm not sick.i don't have a headache.i'm not tired l<br>i'm just sick of all this bs<br>so my dad got a job at the hospital and i'm so fucking happy for him<br>i hate the way you talk to me.	**Low**	**?**	C	**B**	**B**
**B**	i'm not sure why i'm still on my feet.<br>well, this is my first time doing this, so i'm not sure how it's going to work, but i'm goi<br>i have to say, i have had the best weekend ever!friday night i went to a friend's birthd<br>so i'm in a really good mood today... well, i don't know if it's because of the good moo<br>i'm so ready to start this new job	**Neutral**	**?**	B	**C**	**C**
**C**	i'm back from my vacation.it was great.i got to see my family which was really great.i got to s<br>so my mom is on a mission to make me a new pair of jeans.she has a pair of jeans that she lov<br>the man on the moon the man on the moon is a man with a gun in his hand.the man on the mo<br>i'm back from vacation.it's been a good vacation.we went to the beach and went to the amusen<br>this weekend was a blast.i went to a wedding and the reception was so much fun.it was a great	**High**	**?**	A	**A**	**A**

**Set 7**		**Your answer**		**Correct answer**		
**A**	i'm not going to get into why this is happening, because it really doesn't matter.i've b<br>i'm not really a morning person.i'm not sure if it's the sleepiness that i'm used to or t<br>this is the first day i am posting from my new blog.it is a great day and a great time to<br>i have to do some work for the company.i was supposed to do this yesterday, but i di<br>i was thinking about what i should post in my blog today, and i decided to post somet	**Low**	**?**	C	**C**	**C**
**B**	i'm sick of being a loser and not being able to find someone who is willing to be with<br>i'm so sad about my sister's boyfriend.i've been trying to be nice, but i'm just really p<br>i'm so sick of my hair! i hate it!<br>the feeling of being alone in the world.<br>i'm really tired. i think i'll go to sleep.	**Neutral**	**?**	A	**A**	**A**
**C**	i love being able to cook for myself<br>the only real thing in life is the moment of truth<br>it's a beautiful day out in san diego<br>i have a big fat juicy ass.<br>i love the way this man talks	**High**	**?**	B	**B**	**B**

**Set 8**		**Your answer**		**Correct answer**		
	i'm going to start doing my makeup and hair in my bathroom<br>"my mom just said ""i'm glad i'm not the only one who doesn't have a job"" lmao"					

A	it's 11:52.i'm sitting here thinking about how it was so much fun to be in high school<br>it was a great day yesterday.i was in the car and my mom was on the phone with my<br>i have to get my hair done in a week	Low	?	B | B | B
B	my weekend was pretty awesome.i got to hang with my friends, and my boyfriend wa<br>i'm a big fan of the west wing and 24.i'm a big fan of the west wing because of the ch<br>so i'm at the gas station and i'm about to get gas when i see this woman who i know.i<br>this weekend has been pretty awesome.it started off with a big fight with my dad and<br>"life is a highway" i think i'm going to be the first one to say this.i'm in love with this n	Neutral	?	A | A | A
C	it's the last day of the week, and i'm still sick.i'm hoping that tomorrow will be better.i'm not su<br>i'm so fucking tired of being tired.<br>i'm so fucking sick of this shit.<br>i'm a fucking loser. i'm sorry for the last 5 hours.<br>i'm in a bad mood today... i hate when i'm in a bad mood... i'm just a bad mood.	High	?	C | C | C

Set 9		Your answer		Correct answer
A	i'm just a man who's trying to live his life.<br>i'm thinking about getting a new car.i'm thinking about a new car.i'm thinking about a<br>i just wanna go home and relax<br>i love the way you love me.<br>well, the weekend is here.i had a great week with my parents and brothers.i love spe	Low	?	A | A | A
B	i have a horrible feeling about today<br>i'm so tired.i'm so tired of trying to be nice and i'm so tired of being nice.i'm so tired o<br>i'm not really feeling the new year's resolution thing.<br>i'm feeling a bit better today.i was really depressed yesterday.i'm not depressed anyr<br>i just want to die.i just want to die.i just want to die.i just want to die.i just want to die	Neutral	?	C | C | C
C	i'm so tired and i'm so hungry. i'm not getting any sleep tonight.<br>i just wanna make a new tattoo<br>the new blog looks nice.i think i'll stick with it for a while.i was looking at some of the other b<br>well, this is my first attempt at blogging.i have been thinking about it for some time now and i<br>i'm not sure i've ever been so happy to be home.i had such a nice weekend, and it was just wha	High	?	B | B | B

Set 10		Your answer		Correct answer
A	i am going to be at the library until 6pm today.if you would like to stop by and say hi,<br>i'm just trying to get this done<br>i'm so glad i'm not a girl. i'd be so mad if my boyfriend cheated on me.<br>i'm not even going to try to make sense of this, because it's not really worth the effor<br>i can't wait to get my hair done	Low	?	C | C | C
B	i hate the way you make me feel.<br>i'm so tired of people telling me i'm young and i'm still growing. i'm not growing anym<br>i hate being so lonely and having no one to talk to<br>i am so sick of people not liking me.i'm not the kind of person who likes to hate peop<br>i hate when i get really upset and i can't find my phone	Neutral	?	A | A | A
	i'm so ready to see this movie lol			

**C**	i'm thinking about going to the beach this weekend.<br>i'm a man of action... i'll be back in a few minutes<br>i love my life right now!<br>so much to do, so little time.	**High**	**?**		B	**B**	**B**

S2.D. Agreement between annotators and LLM Claude 3.5 (Kappa - Hamming distance)

H1: Human expert 1
H2: Human expert 2
LLM: Claude 3.5

Agreement	**H1/H2**	**H1/LLM**	**H2/LLM**
**Big5**	0.67	0.85	0.71
**Big5 ILT**	0.85	0.91	0.76
**Mental Health**	1	0.95	0.95

S3. Prompt template for Claude annotation

Big Five Personality Claude 3.5 Judging prompts

- 3 levels:

You are an expert in personality psychology tasked with matching sets of text to corresponding levels of <personality> personality. <personality_explanation>. The three levels of <personality> you will be working with are:

1. <negative>: <negative_explanation>
2. Neutral: <neutral_explanation>
3. <positive>: <positive_explanation>

Your task is to analyze these sets and match them to the appropriate level of <personality>.

Set A:
<set_a>
</set_a>

Set B:
<set_b>
</set_b>

Set C:
<set_c>
</set_c>

To complete this task, follow these steps:

1. Carefully read through each set of sentences.
2. Analyze the language, tone, and content of each set.
3. Identify patterns or themes that align with the characteristics of <negative>, Neutral, or <positive> personalities.
4. Match each set to the most appropriate level of <personality>.

Once you have completed your analysis, provide your answer in the following format:
<answer>
[Set letter for <negative>], [Set letter for Neutral], [Set letter for <positive>]
</answer>

Remember to maintain the order of <negative>, Neutral, <positive> in your response. Use only the set names (A, B, or C) without any additional words or explanations.

- Openness:
  - <personality_explanation>: Openness is a personality trait that reflects an individual's curiosity, creativity, and willingness to try new experiences
  - <negative>: Closed to experience
  - <positive>: Open to experience
  - <negative_explanation>: Low openness, characterized by preference for routine, traditional values, and a lack of interest in novelty. Individuals may be more conventional and resistant to change
  - <neutral_explanation>: Moderate openness, showing a balance between seeking new experiences and maintaining routine. These individuals may be open to new ideas but also value stability and tradition
  - <positive_explanation>: High openness, characterized by curiosity, creativity, and a strong preference for novelty and variety. Individuals are often imaginative, open-minded, and willing to explore new ideas and experiences
- Conscientiousness:
  - <personality_explanation>: onscientiousness is a personality trait characterized by being

organized, responsible, hardworking, and goal-oriented. People high in conscientiousness tend to be efficient, disciplined, and detail-oriented, while those low in conscientiousness may be more spontaneous, disorganized, or careless

- <negative>: Unconscientious
- <positive>: Conscientious
- <negative_explanation>: Low conscientiousness, characterized by disorganization, carelessness, and lack of reliability/planning
- <neutral_explanation>: Moderate conscientiousness, a mix of conscientious and unconscientious behaviors, or statements that don't strongly indicate either extreme
- <positive_explanation>: High conscientiousness, demonstrating organization, responsibility, attention to detail, and goal-oriented behavior

- Extraversion:
  - <personality_explanation>: Extroversion is a personality trait typically characterized by outgoingness, high energy, and/or talkativeness
  - <negative>: Introversion
  - <positive>: Extroversion
  - <negative_explanation>: Characterized by a preference for solitude, quiet environments, and internal reflection
  - <neutral_explanation>: Balanced between introverted and extraverted tendencies, comfortable in both social and solitary situations
  - <positive_explanation>: Characterized by outgoing behavior, enjoyment of social interactions, and high energy in social settings
- Agreeableness:
  - <personality_explanation>: Agreeableness is a personality trait characterized by attributes such as kindness, cooperativeness, and empathy
  - <negative>: Antagonistic
  - <positive>: Agreeable
  - <negative_explanation>: Low agreeableness, characterized by hostility, criticism, and lack of cooperation
  - <neutral_explanation>: Moderate agreeableness, showing neither strong antagonism nor high agreeableness
  - <positive_explanation>: High agreeableness, demonstrating kindness, cooperation, and empathy
- Neuroticism:
  - <personality_explanation>: Neuroticism is a personality trait characterized by emotional instability, anxiety, moodiness, and a propensity for experiencing negative emotions. People high in neuroticism tend to be more susceptible to stress, worry, and feelings of sadness or irritability
  - <negative>: Emotionally stable
  - <positive>: Neuroticism
  - <negative_explanation>: characterized by calmness, emotional resilience, and the ability to handle stress well
  - <neutral_explanation>: showing a balance between emotional stability and neuroticism, with moderate emotional responses
  - <positive_explanation>: marked by a tendency towards negative emotions, anxiety, and emotional instability

- 5 levels:

You are an expert in personality psychology tasked with matching sets of text to corresponding levels of <personality> personality. <personality_explanation>. The five levels of <personality> you will be working with are:

1. Highly <negative>
2. Moderately <negative>
3. Neutral
4. Moderately <positive>
5. Highly <positive>

<levels_explanation>

Your task is to analyze these sets and match them to the appropriate level of <personality>.

Set A:
<set_a>
</set_a>

Set B:
<set_b>
</set_b>

Set C:
<set_c>
</set_c>

Set D:
<set_d>
</set_d>

Set E:
<set_e>
</set_e>

To complete this task, follow these steps:

1. Carefully read through each set of sentences.
2. Analyze the language, tone, and content of each set.
3. Identify patterns or themes that align with the characteristics of Highly <negative>, Moderately <negative>, Neutral, Moderately <positive>, or Highly <positive> personalities.
4. Match each set to the most appropriate level of Neuroticism.

Once you have completed your analysis, provide your answer in the following format:
<answer>
[Set letter for Highly <negative>], [Set letter for Moderately <negative>], [Set letter for Neutral], [Set letter for Moderately <positive>], [Set letter for Highly <positive>]
</answer>

Remember to maintain the order of Highly <negative>, Moderately <negative>, Neutral, Moderately <positive>, Highly <positive> in your response. Use only the set names (A, B, C, D, or E) without any additional words or explanations.

- Openness:
  - <personality_explanation>: Openness is a personality trait that reflects an individual's curiosity, creativity, and willingness to try new experiences
  - <negative> Closed to experience
  - <positive> Open to experience

    - <levels_explanation>: In which, Highly Closed to experience is characterized by low openness, characterized by preference for routine, traditional values, and a lack of interest in novelty. Individuals who are closed to experience may be more conventional and resistant to change. Highly Open to experience is characterized by curiosity, creativity, and a strong preference for novelty and variety. Individuals who are open to experience often imaginative, open-minded, and willing to explore new ideas and experiences. Neutral is moderate openness, showing a balance between seeking new experiences and maintaining routine. These individuals may be open to new ideas but also value stability and tradition.
- Conscientiousness:
    - <personality_explanation>: Conscientiousness is a personality trait characterized by being organized, responsible, hardworking, and goal-oriented. People high in conscientiousness tend to be efficient, disciplined, and detail-oriented, while those low in conscientiousness may be more spontaneous, disorganized, or careless
    - <negative> Unconscientious
    - <positive> Conscientiousness
    - <levels_explanation>: In which, Conscientiousness is demonstrated through organization, responsibility, attention to detail, and goal-oriented behavior. Unconscientious is disorganization, carelessness, and lack of reliability/planning. Neutral is a mix of conscientious and unconscientious behaviors, or statements that don't strongly indicate either extreme.
- Extraversion:
    - <personality_explanation>: Extroversion is a personality trait typically characterized by outgoingness, high energy, and/or talkativeness
    - <negative> Introversion
    - <positive> Extroversion
    - <levels_explanation>: In which, Extroversion is characterized by outgoing behavior, enjoyment of social interactions, and high energy in social settings. Introversion is characterized by a preference for solitude, quiet environments, and internal reflection. Neutral is balanced between introverted and extraverted tendencies, comfortable in both social and solitary situations.
- Agreeableness:
    - <personality_explanation>: Agreeableness is a personality trait characterized by attributes such as kindness, cooperativeness, and empathy
    - <negative> Antagonistic
    - <positive> Agreeable
    - <levels_explanation>: In which, Antagonistic behavior is characterized by low agreeableness, including hostility, criticism, and a lack of cooperation. In contrast, Agreeable behavior is marked by kindness, cooperation, and empathy. Neutral behavior does not strongly exhibit either antagonism or high agreeableness.
- Neuroticism:
    - <personality_explanation>: Neuroticism is a personality trait characterized by emotional instability, anxiety, moodiness, and a propensity for experiencing negative emotions. People high in neuroticism tend to be more susceptible to stress, worry, and feelings of sadness or irritability
    - <negative> Emotionally Stable
    - <positive> Neuroticism
    - <levels_explanation>: In which, Highly Emotionally Stable is characterized by calmness, emotional resilience, and the ability to handle stress well. Neuroticism is marked by a tendency towards negative emotions, anxiety, and emotional instability. Neutral is showing a balance between emotional stability and neuroticism, with moderate emotional responses.

Mental Health Claude 3.5 Judging prompts

- 5 levels:

```
You are an expert in mental health psychology tasked with matching sets of text to corresponding levels of <mental_heath_variable>. <mental_heath_variable_explanation>. The five levels of <mental_heath_variable> you will be working with are:

1. Low <mental_heath_variable>: <low_explanation>
2. Moderately Low <mental_heath_variable>: <moderately_low_explanation>
3. Neutral: <neutral_explanation>
4. Moderately High <mental_heath_variable>: <moderately_high_explanation>
5. High <mental_heath_variable>: <high_explanation>

Your task is to analyze these sets and match them to the appropriate level of <mental_heath_variable>.

Set A:
<set_a>
</set_a>

Set B:
<set_b>
</set_b>

Set C:
<set_c>
</set_c>

Set D:
<set_d>
</set_d>

Set E:
<set_e>
</set_e>


To complete this task, follow these steps:

1. Carefully read through each set of sentences.
2. Analyze the language, tone, and content of each set.
3. Identify patterns or themes that align with the characteristics of Low <mental_heath_variable>, Moderately Low <mental_heath_variable>, Neutral, Moderately High <mental_heath_variable>, or High <mental_heath_variable>.
4. Match each set to the most appropriate level of <mental_heath_variable>.

Once you have completed your analysis, provide your answer in the following format:
<answer>
[Set letter for Low <mental_heath_variable>], [Set letter for Moderately Low <mental_heath_variable>], [Set letter for Neutral], [Set letter for Moderately High <mental_heath_variable>], [Set letter for High <mental_heath_variable>]
</answer>

Remember to maintain the order of Low <mental_heath_variable>, Moderately Low <mental_heath_variable>, Neutral, Moderately High <mental_heath_variable>, High <mental_heath_variable> in your response. Use only the set names (A, B, C, D, or E) without any additional words or explanations.
```

- Life-satisfaction:
    - <mental_heath_variable_explanation>: Life satisfaction refers to an individual's overall assessment of their quality of life according to their chosen criteria. It encompasses emotional well-being, fulfillment, and contentment

- <low_explanation>: this level reflects strong dissatisfaction or unhappiness with life. Individuals express negative emotions, frustration, and pessimism. Examples include feeling hopeless, unfulfilled, or that life is meaningless
- <moderately_low_explanation>: this level indicates some dissatisfaction, though not as severe as the lowest level. People may recognize a few positives, but negatives dominate. They often feel unfulfilled but not entirely hopeless
- <neutral_explanation>: This means an individual neither feels particularly satisfied nor dissatisfied. Life is viewed as adequate or average, without strong emotions. They may describe life as fine or okay
- <moderatelt_high_explanation>: individuals at this level feel generally content with life, though there may still be areas to improve. Positives outweigh negatives, and there\u2019s a sense of fulfillment. Life is good but not without challenges
- <high_explanation>: this reflects a deep sense of joy, contentment, and fulfillment. Individuals feel life exceeds their expectations, with little or no dissatisfaction. They express happiness, gratitude, and pride in their life

- Depression:
  - <mental_heath_variable_explanation>: Depression is a mental health condition characterized by persistent feelings of sadness, hopelessness, and a lack of interest or pleasure in daily activities. It can affect a person's thoughts, behavior, and overall well-being
  - <low_explanation>: May include positive emotions, optimism, energy, and engagement in activities. The individual may still experience mild low moods but remains mostly positive and functional
  - <moderately_low_explanation>: May include some periods of sadness or low mood, but there is still hope and energy for activities. The individual may experience occasional dissatisfaction but remains mostly positive
  - <neutral_explanation>: Involves a balanced mix of positive and negative emotions. The individual feels neither particularly high nor low, with no strong leanings toward either side
  - <moderatelt_high_explanation>: Involves a noticeable increase in negative emotions and disengagement. The individual may experience more frequent low periods but still finds some motivation and engagement
  - <high_explanation>: Involves predominantly negative emotions and a sense of disconnection or dissatisfaction with daily life. The individual may find little fulfillment or engagement in activities

S4. Comparison of the PsychAdapter approach to Prompt Engineering

This experiment compares the PsychAdapter approach to prompt engineering on two model architectures: Gemma-2B and LLaMA3-8B. Texts generated by both methods were automatically evaluated using Claude 3.5 Sonnet, and the resulting confusion matrices between the intended and Claude inferred personality traits were reported. The evaluation covered five personality traits, with performance measured by accuracy averaged across all results. For the prompt engineering baseline, we used Gemma-2B-Instruct [1] and LLaMA3-8B-Instruct [2], as these variants exhibit stronger instruction-following capabilities. The generation prompt (shown below) defines the maximum, minimum, and neutral levels of each psychological trait, expecting the model to interpolate the intermediate fine-grained levels (e.g., levels 2 and 4). This setup directly tests prompt engineering's ability to achieve fine-grained control over a continuous input variable against PsychAdapter's approach. For the smaller model (Gemma-2B), PsychAdapter achieved an average accuracy of 0.772, significantly outperforming prompt engineering (0.532). As shown in Figure S1, the prompt engineering approach struggled particularly with interpolated and neutral trait levels. For the larger model (LLaMA3-8B), PsychAdapter obtained an average accuracy of 0.788, nominally higher than prompt engineering (0.768).

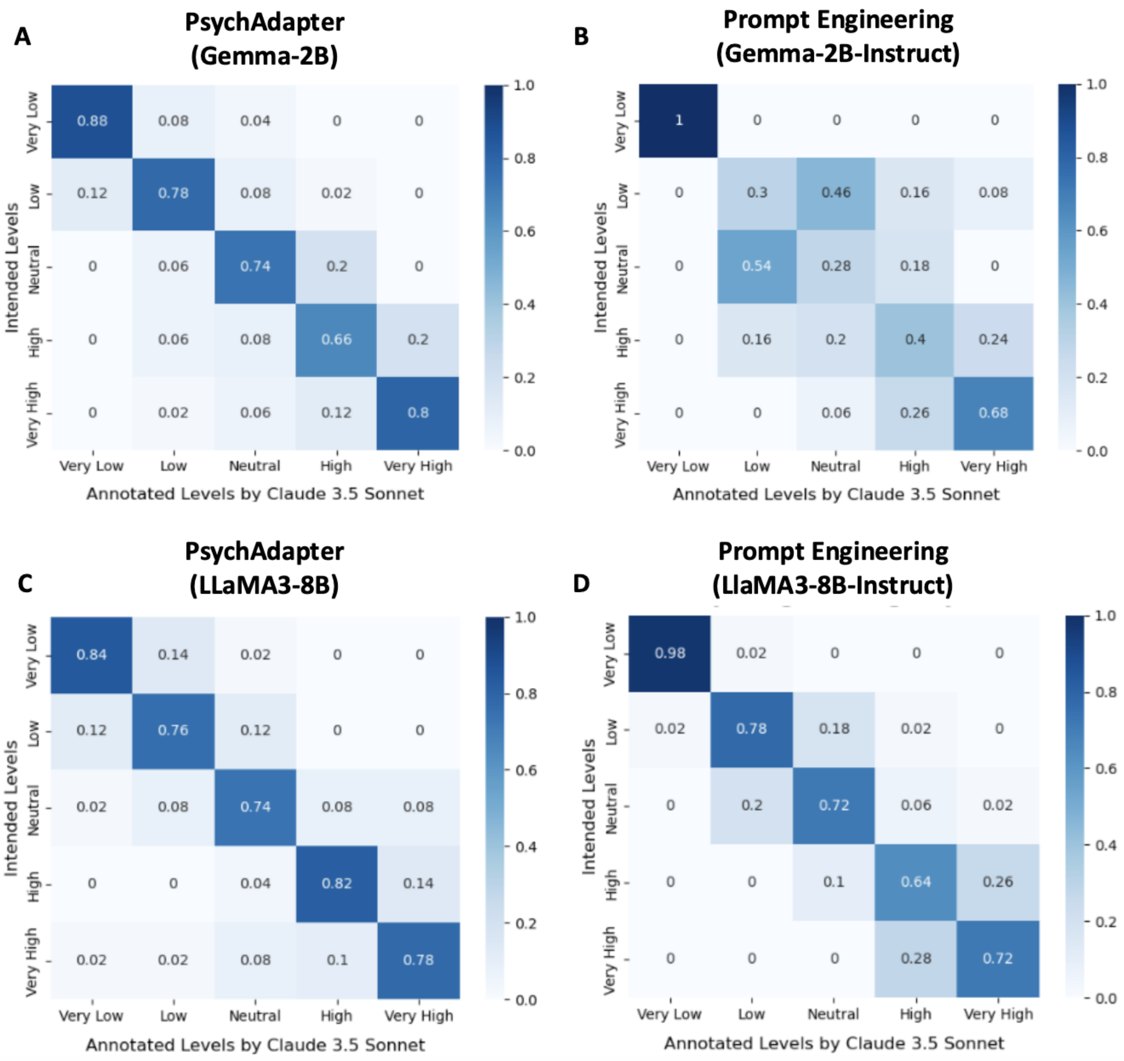

Fig. S1. Confusion matrixes comparing PsychAdapter with Prompt Engineering, across two model sizes: Gemma-2B and LLaMA3-8B.

Prompt used for prompt engineering approach:

> You are an expert in personality psychology. Your task is to generate social media posts/micro blogs written by a person with a specific personality profile. The personality trait you will simulate is *{personality}*. The score of *{personality}* ranges from 1 to 5, where 1 is the low end of the scale: Very negative, 3 is Neutral, and 5 is Very positive and the high end of the scale. Write 10 examples of social media posts/micro blogs illustrating a level of *{personality}* of *{value}*. Do not write any other words, just write the examples, starting each example with a hyphen.

where *{personality}* takes one of the five Big Five traits - Openness, Conscientiousness, Extraversion, Acceptability or Neuroticism - and *{value}* takes a discrete level from 1 to 5.

REFERENCES

1. G. Team, M. Rivière, S. Pathak, *et al.*, “Gemma 2: Improving open language models at a practical size,” arXiv preprint arXiv:2408.00118 (2024).
2. I. Meta Platforms, “Meta llama 3 – 8b: Instruction-tuned variant,” Model card available at Hugging Face and Meta website (2024). Release date April 18, 2024. Model ID “meta-llama/Meta-Llama-3-8B-Instruct”.
